# Supplementary material for: Association of VDR Polymorphisms (FokI, ApaI, and TaqI) with Susceptibility to Lumbar Disc Herniation: Systematic Review, Meta-Analysis, Trial Sequential Analysis, and Transcriptional Prediction
Source: Medicina (Kaunas). 2025 May 12;61(5):882. doi: 10.3390/medicina61050882 (PMC12113608; doi:10.3390/medicina61050882)
Supplement: Supplementary file 1 [file medicina-61-00882-s001.zip › Supplementary File S1.pdf]

Study or Subgroup	Case Events	Case Total	Control Events	Control Total	Weight	Odds Ratio M-H, Random, 95% CI
Colombini, 2014	64	178	163	440	14.3%	0.95 [0.66, 1.37]
Colombini, 2015	27	96	96	254	11.0%	0.64 [0.39, 1.07]
Gaydarski, 2023	56	120	31	120	10.4%	2.51 [1.46, 4.33]
Hekimoğlu, 2020	36	144	28	162	10.2%	1.60 [0.92, 2.78]
Li, 2018	99	240	112	240	14.3%	0.80 [0.56, 1.15]
Sansoni, 2016	70	220	74	220	13.4%	0.92 [0.62, 1.37]
Withanage, 2018	18	102	34	136	8.7%	0.64 [0.34, 1.22]
Yang, 2019	275	532	477	970	17.7%	1.11 [0.90, 1.37]
**Total (95% CI)**		**1632**		**2542**	**100.0%**	**1.03 [0.80, 1.32]**
Total events	645		1015			

Heterogeneity: $Tau^2 = 0.08$; $Chi^2 = 20.77$, df = 7 (P = 0.004); $I^2 = 66\%$
Test for overall effect: Z = 0.19 (P = 0.85)

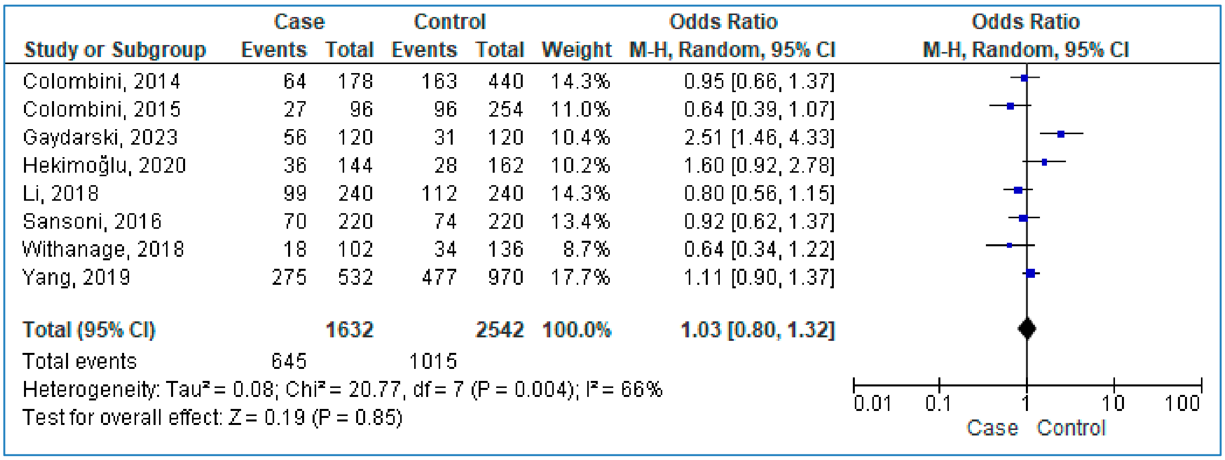

Figure S1: Forest plot of the association of *FokI* polymorphism and the risk of lumbar disk herniation in allelic model

Study or Subgroup	Case Events	Case Total	Control Events	Control Total	Weight	Odds Ratio M-H, Fixed, 95% CI
Colombini, 2014	12	49	32	121	14.4%	0.90 [0.42, 1.94]
Colombini, 2015	4	29	20	71	10.4%	0.41 [0.13, 1.32]
Gaydarski, 2023	8	20	0	29	0.3%	40.12 [2.15, 749.75]
Hekimoğlu, 2020	1	38	2	57	1.6%	0.74 [0.07, 8.50]
Li, 2018	23	67	23	54	17.3%	0.70 [0.34, 1.47]
Sansoni, 2016	13	66	10	56	9.0%	1.13 [0.45, 2.81]
Withanage, 2018	1	35	4	42	3.7%	0.28 [0.03, 2.62]
Yang, 2019	76	143	126	260	43.4%	1.21 [0.80, 1.82]
**Total (95% CI)**		**447**		**690**	**100.0%**	**1.04 [0.79, 1.38]**
Total events	138		217			

Heterogeneity: Chi² = 11.56, df = 7 (P = 0.12); I² = 39%
Test for overall effect: Z = 0.30 (P = 0.77)

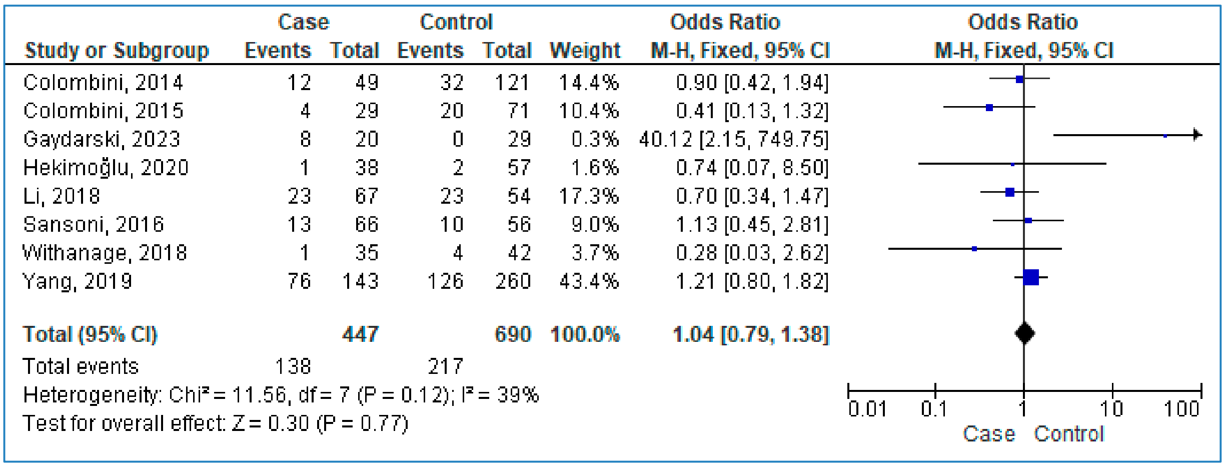

Figure S2: Forest plot of the association of *FokI* polymorphism and the risk of lumbar disk herniation in homozygous model

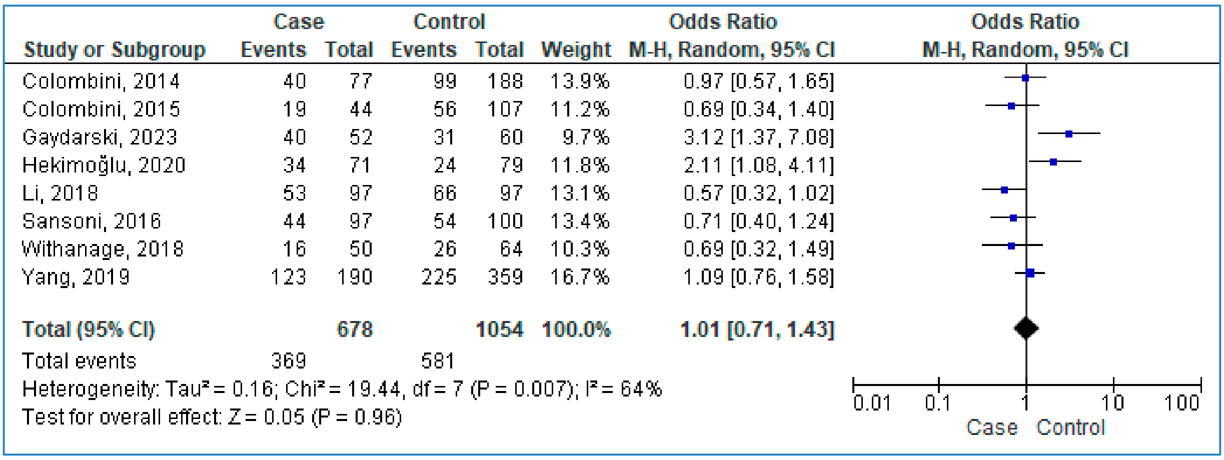

Study or Subgroup	Case Events	Case Total	Control Events	Control Total	Weight	Odds Ratio M-H, Random, 95% CI
Colombini, 2014	40	77	99	188	13.9%	0.97 [0.57, 1.65]
Colombini, 2015	19	44	56	107	11.2%	0.69 [0.34, 1.40]
Gaydarski, 2023	40	52	31	60	9.7%	3.12 [1.37, 7.08]
Hekimoğlu, 2020	34	71	24	79	11.8%	2.11 [1.08, 4.11]
Li, 2018	53	97	66	97	13.1%	0.57 [0.32, 1.02]
Sansoni, 2016	44	97	54	100	13.4%	0.71 [0.40, 1.24]
Withanage, 2018	16	50	26	64	10.3%	0.69 [0.32, 1.49]
Yang, 2019	123	190	225	359	16.7%	1.09 [0.76, 1.58]
**Total (95% CI)**		**678**		**1054**	**100.0%**	**1.01 [0.71, 1.43]**
Total events	369		581			

Heterogeneity: $Tau^2 = 0.16$; $Chi^2 = 19.44$, df = 7 (P = 0.007); $I^2 = 64\%$

Test for overall effect: Z = 0.05 (P = 0.96)

Figure S3: Forest plot of the association of *FokI* polymorphism and the risk of lumbar disk herniation in heterozygous model

Study or Subgroup	Case Events	Case Total	Control Events	Control Total	Weight	Odds Ratio M-H, Random, 95% CI
Colombini, 2014	52	89	131	220	13.9%	0.95 [0.58, 1.57]
Colombini, 2015	23	48	76	127	11.5%	0.62 [0.32, 1.20]
Gaydarski, 2023	48	60	31	60	9.7%	3.74 [1.66, 8.41]
Hekimoğlu, 2020	35	72	26	81	11.7%	2.00 [1.04, 3.86]
Li, 2018	76	120	89	120	13.1%	0.60 [0.35, 1.05]
Sansoni, 2016	57	110	64	110	13.4%	0.77 [0.45, 1.32]
Withanage, 2018	17	51	30	68	10.4%	0.63 [0.30, 1.35]
Yang, 2019	199	266	351	485	16.2%	1.13 [0.81, 1.59]
**Total (95% CI)**		**816**		**1271**	**100.0%**	**1.02 [0.71, 1.46]**
Total events	507		798			

Heterogeneity: Tau² = 0.18; Chi² = 22.60, df = 7 (P = 0.002); I² = 69%

Test for overall effect: Z = 0.10 (P = 0.92)

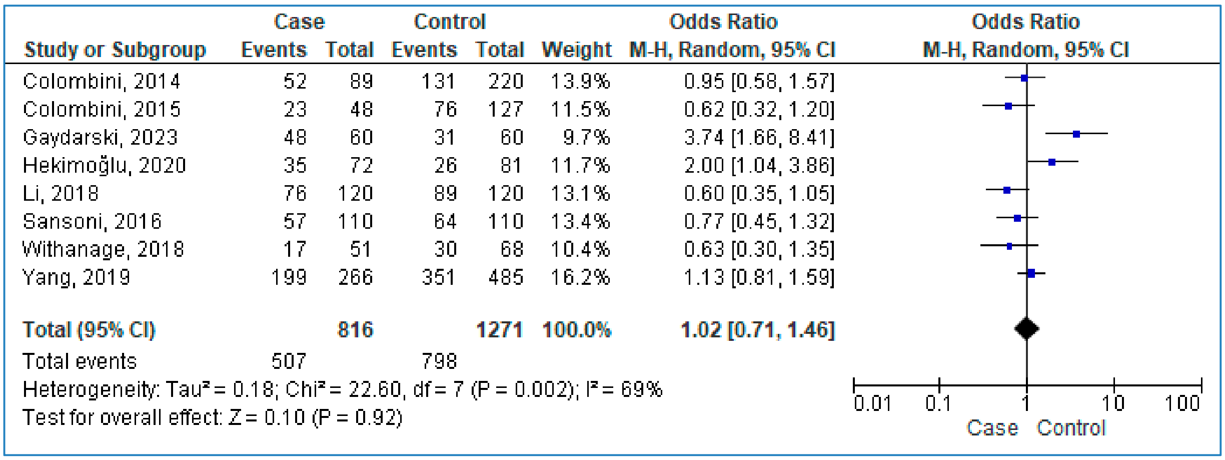

Figure S4: Forest plot of the association of *FokI* polymorphism and the risk of lumbar disk herniation in dominant model

Study or Subgroup	Case Events	Case Total	Control Events	Control Total	Weight	Odds Ratio M-H, Fixed, 95% CI
Colombini, 2014	12	89	32	220	13.0%	0.92 [0.45, 1.87]
Colombini, 2015	4	48	20	127	8.2%	0.49 [0.16, 1.50]
Gaydarski, 2023	8	60	0	60	0.4%	19.59 [1.10, 347.61]
Hekimoğlu, 2020	1	72	2	81	1.5%	0.56 [0.05, 6.27]
Li, 2018	23	120	23	120	15.1%	1.00 [0.53, 1.90]
Sansoni, 2016	13	110	10	110	7.2%	1.34 [0.56, 3.20]
Withanage, 2018	1	51	4	68	2.7%	0.32 [0.03, 2.95]
Yang, 2019	76	266	126	485	51.9%	1.14 [0.82, 1.59]
**Total (95% CI)**		**816**		**1271**	**100.0%**	**1.08 [0.85, 1.38]**
Total events	138		217			

Heterogeneity: Chi² = 7.86, df = 7 (P = 0.34); I² = 11%
Test for overall effect: Z = 0.64 (P = 0.52)

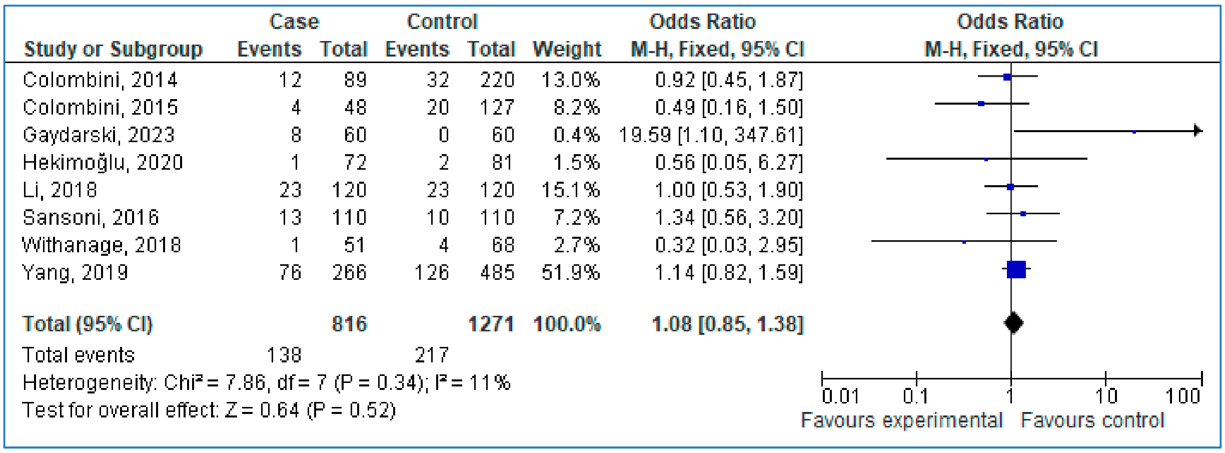

Figure S5: Forest plot of the association of *FokI* polymorphism and the risk of lumbar disk herniation in recessive model

Study or Subgroup	Case Events	Case Total	Control Events	Control Total	Weight	Odds Ratio M-H, Fixed, 95% CI
Colombini, 2016	73	176	212	504	24.8%	0.98 [0.69, 1.38]
Li, 2018	167	240	160	240	18.8%	1.14 [0.78, 1.68]
Yang, 2019	156	532	291	970	56.3%	0.97 [0.77, 1.22]
**Total (95% CI)**		**948**		**1714**	**100.0%**	**1.00 [0.84, 1.19]**
Total events	396		663			

Heterogeneity: Chi² = 0.56, df = 2 (P = 0.75); I² = 0%
Test for overall effect: Z = 0.04 (P = 0.97)

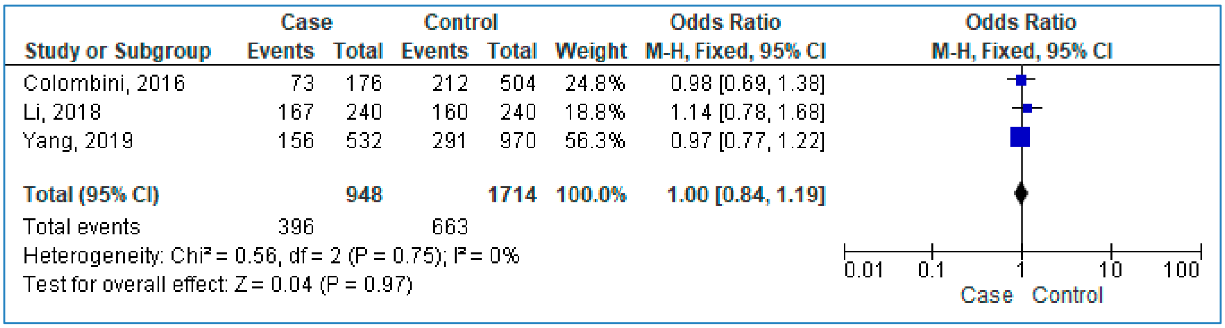

Figure S6: Forest plot of the association of *ApaI* polymorphism and the risk of lumbar disk herniation in allelic model

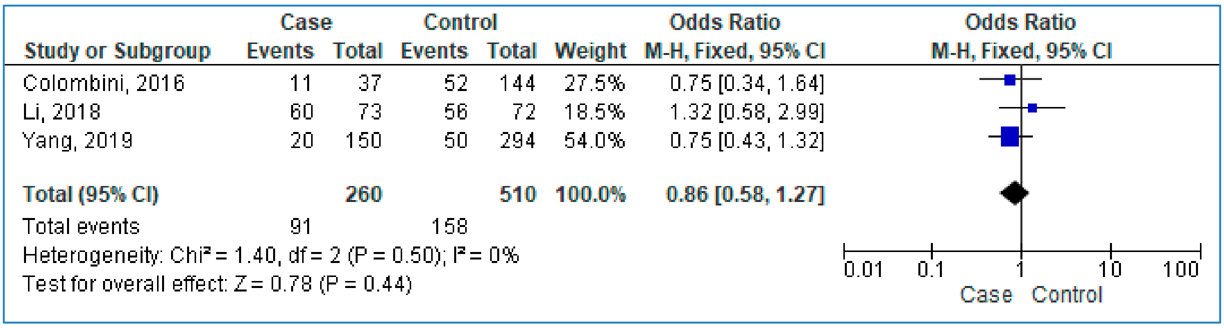

Figure S7: Forest plot of the association of *ApaI* polymorphism and the risk of lumbar disk herniation in homozygous model

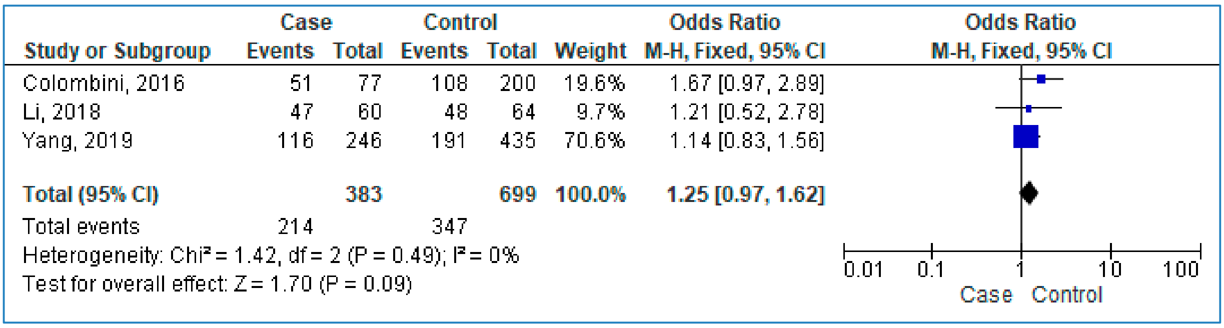

Figure S8: Forest plot of the association of *ApaI* polymorphism and the risk of lumbar disk herniation in heterozygous model

Study or Subgroup	Case Events	Case Total	Control Events	Control Total	Weight	Odds Ratio M-H, Fixed, 95% CI
Colombini, 2016	62	88	160	252	20.5%	1.37 [0.81, 2.32]
Li, 2018	107	120	104	120	9.5%	1.27 [0.58, 2.76]
Yang, 2019	136	266	241	485	70.0%	1.06 [0.79, 1.43]
**Total (95% CI)**		**474**		**857**	**100.0%**	**1.14 [0.89, 1.46]**
Total events	305		505			

Heterogeneity: Chi² = 0.78, df = 2 (P = 0.68); I² = 0%

Test for overall effect: Z = 1.06 (P = 0.29)

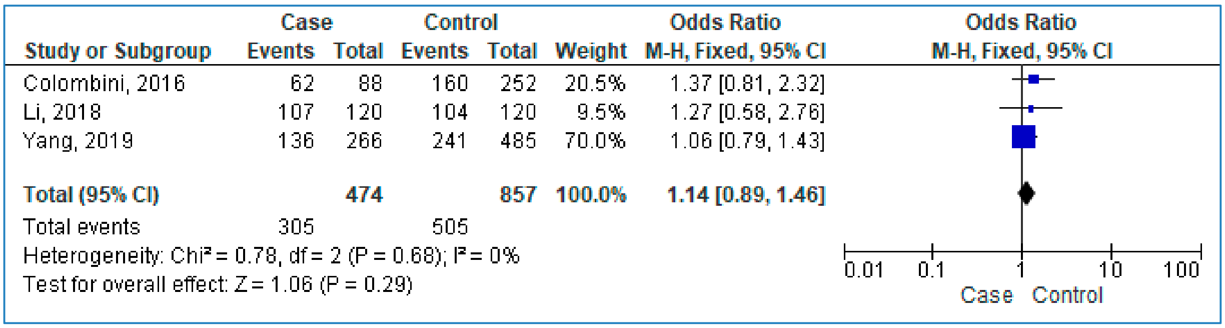

Figure S9: Forest plot of the association of *ApaI* polymorphism and the risk of lumbar disk herniation in dominant model

Study or Subgroup	Case Events	Case Total	Control Events	Control Total	Weight	Odds Ratio M-H, Fixed, 95% CI
Colombini, 2016	11	88	52	252	27.9%	0.55 [0.27, 1.11]
Li, 2018	60	120	56	120	33.2%	1.14 [0.69, 1.90]
Yang, 2019	20	266	50	485	38.9%	0.71 [0.41, 1.22]
**Total (95% CI)**		**474**		**857**	**100.0%**	**0.81 [0.59, 1.11]**
Total events	91		158			

Heterogeneity: $Chi^2 = 3.19$, $df = 2$ ($P = 0.20$); $I^2 = 37\%$

Test for overall effect: $Z = 1.30$ ($P = 0.19$)

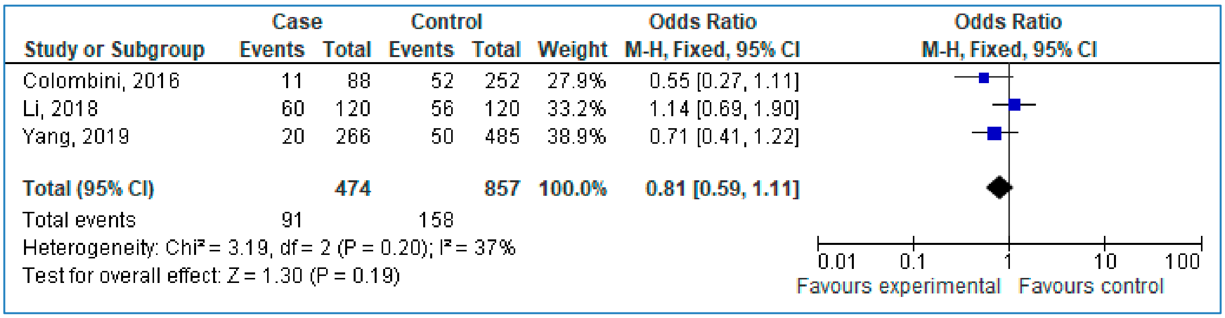

Figure S10: Forest plot of the association of *ApaI* polymorphism and the risk of lumbar disk herniation in recessive model

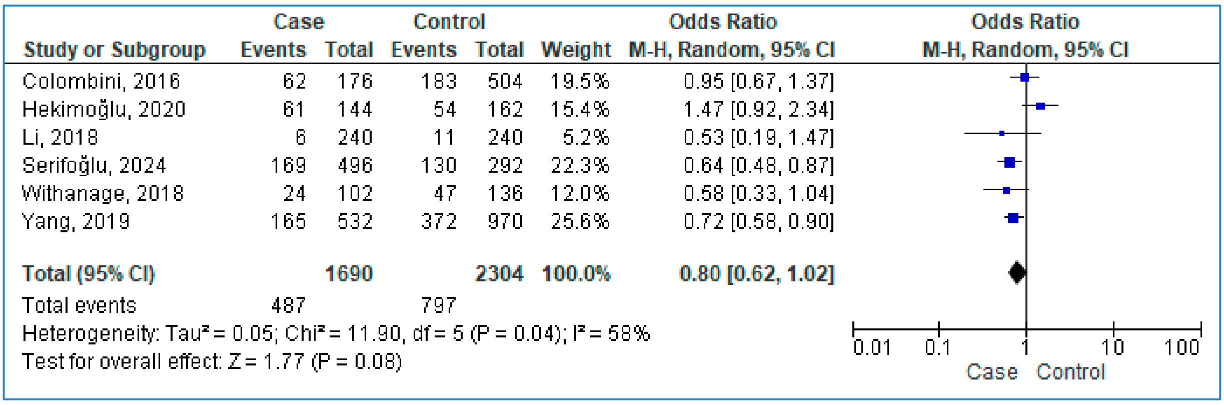

Figure S11: Forest plot of the association of *TaqI* polymorphism and the risk of lumbar disk herniation in allelic model

Study or Subgroup	Case Events	Case Total	Control Events	Control Total	Weight	Odds Ratio M-H, Fixed, 95% CI
Colombini, 2016	11	48	37	143	15.3%	0.85 [0.39, 1.84]
Hekimoğlu, 2020	8	27	12	51	6.3%	1.37 [0.48, 3.91]
Li, 2018	0	114	0	109		Not estimable
Serifoğlu, 2024	28	135	29	74	31.8%	0.41 [0.22, 0.76]
Withanage, 2018	4	35	4	29	4.1%	0.81 [0.18, 3.55]
Yang, 2019	17	135	63	239	42.5%	0.40 [0.22, 0.72]
**Total (95% CI)**		**494**		**645**	**100.0%**	**0.55 [0.39, 0.77]**
Total events	68		145			

Heterogeneity: $Chi^2 = 6.40$, df = 4 (P = 0.17); $I^2 = 37\%$
Test for overall effect: Z = 3.45 (P = 0.0006)

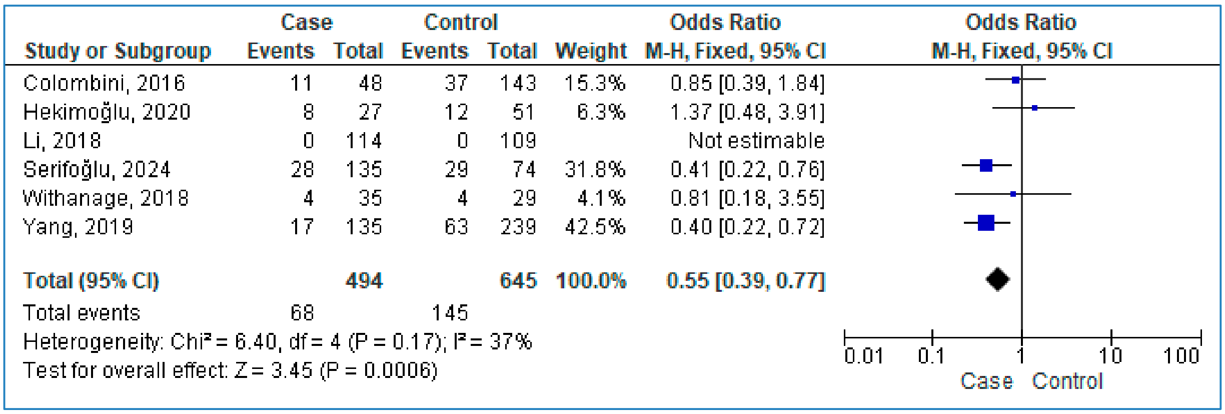

Figure S12: Forest plot of the association of *TaqI* polymorphism and the risk of lumbar disk herniation in homozygous model

Study or Subgroup	Case Events	Case Total	Control Events	Control Total	Weight	Odds Ratio M-H, Random, 95% CI
Colombini, 2016	40	77	109	215	18.4%	1.05 [0.62, 1.77]
Hekimoğlu, 2020	45	64	30	69	15.3%	3.08 [1.50, 6.31]
Li, 2018	6	120	11	120	11.1%	0.52 [0.19, 1.46]
Serifoğlu, 2024	113	220	72	117	19.4%	0.66 [0.42, 1.04]
Withanage, 2018	16	47	39	64	14.3%	0.33 [0.15, 0.73]
Yang, 2019	131	249	246	422	21.4%	0.79 [0.58, 1.09]
**Total (95% CI)**		**777**		**1007**	**100.0%**	**0.84 [0.52, 1.34]**
Total events	351		507			

Heterogeneity: $Tau^2 = 0.24$; $Chi^2 = 20.73$, df = 5 (P = 0.0009); $I^2 = 76\%$

Test for overall effect: Z = 0.75 (P = 0.46)

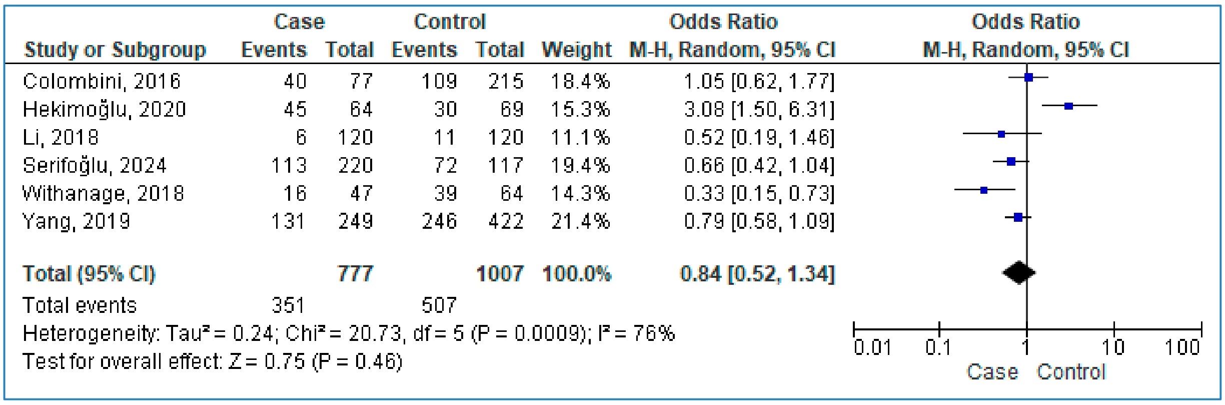

Figure S13: Forest plot of the association of *TaqI* polymorphism and the risk of lumbar disk herniation in heterozygous model

Study or Subgroup	Case Events	Case Total	Control Events	Control Total	Weight	Odds Ratio M-H, Random, 95% CI
Colombini, 2016	51	88	146	252	18.6%	1.00 [0.61, 1.64]
Hekimoğlu, 2020	53	72	42	81	15.3%	2.59 [1.31, 5.12]
Li, 2018	6	120	11	120	10.3%	0.52 [0.19, 1.46]
Serifoğlu, 2024	141	248	101	146	19.7%	0.59 [0.38, 0.90]
Withanage, 2018	20	51	43	68	14.2%	0.38 [0.18, 0.79]
Yang, 2019	148	266	309	485	21.9%	0.71 [0.53, 0.97]
**Total (95% CI)**		**845**		**1152**	**100.0%**	**0.79 [0.51, 1.21]**
Total events	419		652			

Heterogeneity: Tau² = 0.20; Chi² = 19.11, df = 5 (P = 0.002); I² = 74%

Test for overall effect: Z = 1.08 (P = 0.28)

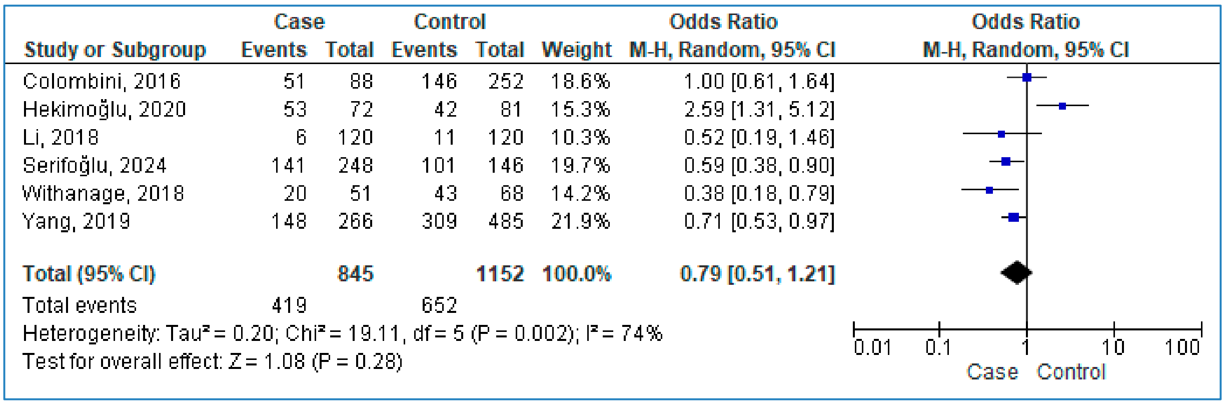

Figure S14: Forest plot of the association of *TaqI* polymorphism and the risk of lumbar disk herniation in dominant model

Study or Subgroup	Case Events	Case Total	Control Events	Control Total	Weight	Odds Ratio M-H, Fixed, 95% CI
Colombini, 2016	11	88	37	252	16.1%	0.83 [0.40, 1.71]
Hekimoğlu, 2020	8	72	12	81	9.6%	0.72 [0.28, 1.87]
Li, 2018	0	120	0	120		Not estimable
Serifoğlu, 2024	28	248	29	146	31.1%	0.51 [0.29, 0.90]
Withanage, 2018	4	51	4	68	3.0%	1.36 [0.32, 5.73]
Yang, 2019	17	266	63	485	40.1%	0.46 [0.26, 0.80]
**Total (95% CI)**		**845**		**1152**	**100.0%**	**0.59 [0.43, 0.81]**
Total events	68		145			

Heterogeneity: Chi² = 3.36, df = 4 (P = 0.50); I² = 0%

Test for overall effect: Z = 3.28 (P = 0.001)

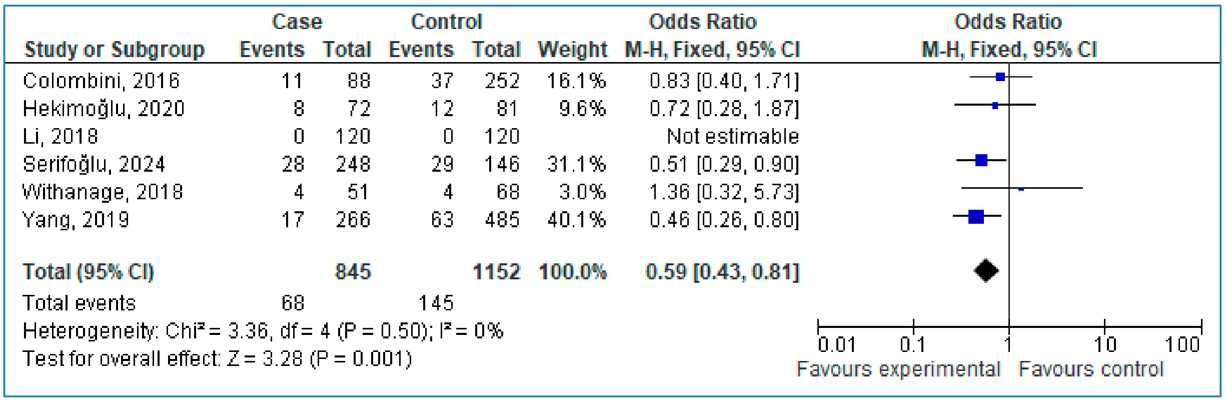

Figure S15: Forest plot of the association of *TaqI* polymorphism and the risk of lumbar disk herniation in recessive model
