# Supplementary material for: Association of VDR Polymorphisms (FokI, ApaI, and TaqI) with Susceptibility to Lumbar Disc Herniation: Systematic Review, Meta-Analysis, Trial Sequential Analysis, and Transcriptional Prediction
Source: Medicina (Kaunas). 2025 May 12;61(5):882. doi: 10.3390/medicina61050882 (PMC12113608; doi:10.3390/medicina61050882)
Supplement: Supplementary file 1 [file medicina-61-00882-s001.zip › Supplementary File S2.pdf]

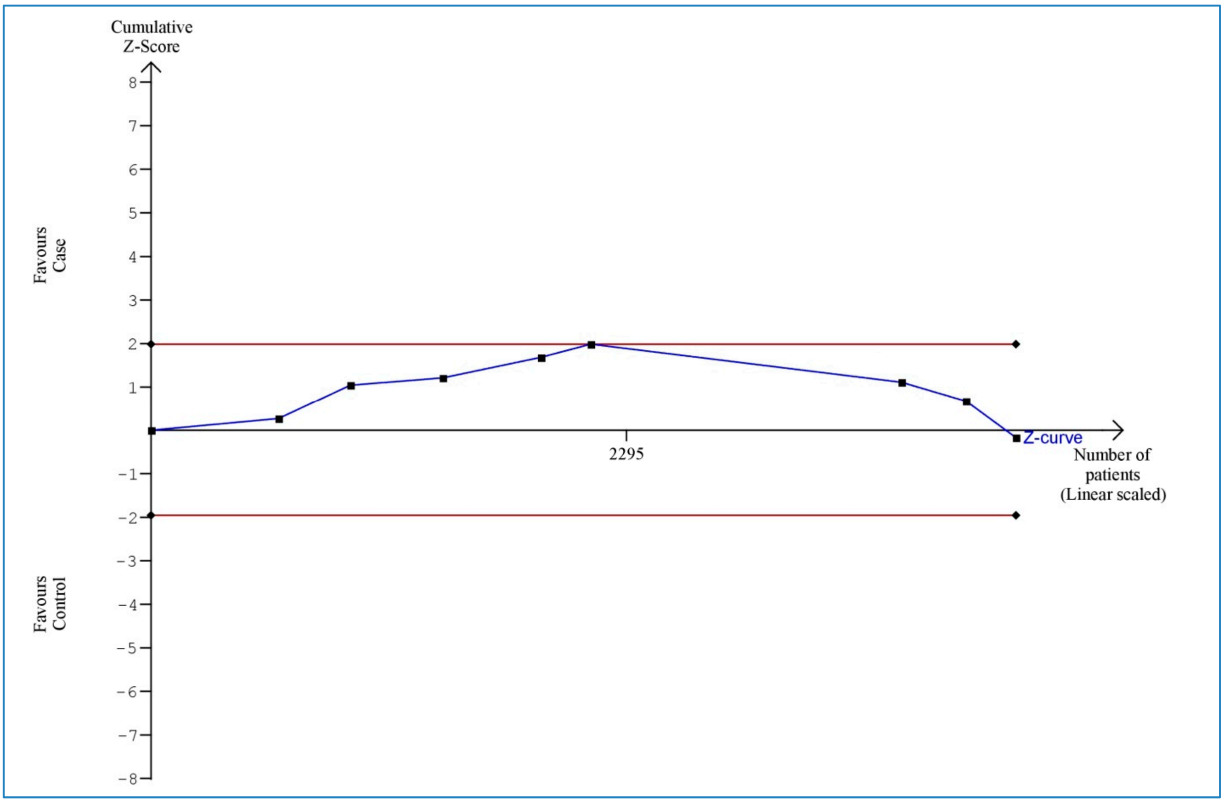

Figure S1: Trial sequential analysis of the association of *FokI* polymorphism and the risk of lumbar disk herniation in allelic model (D^2: 72%)

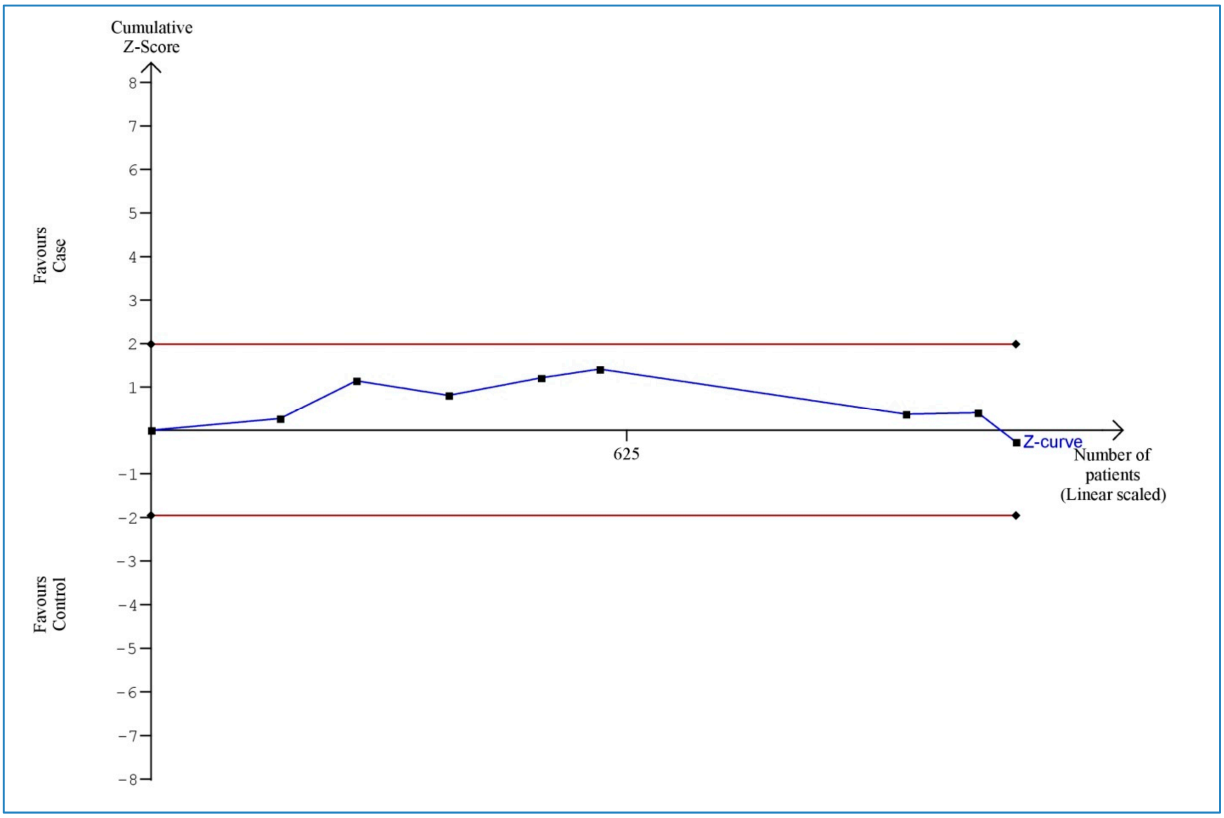

Figure S2: Trial sequential analysis of the association of *FokI* polymorphism and the risk of lumbar disk herniation in homozygous model (D^2: 59%)

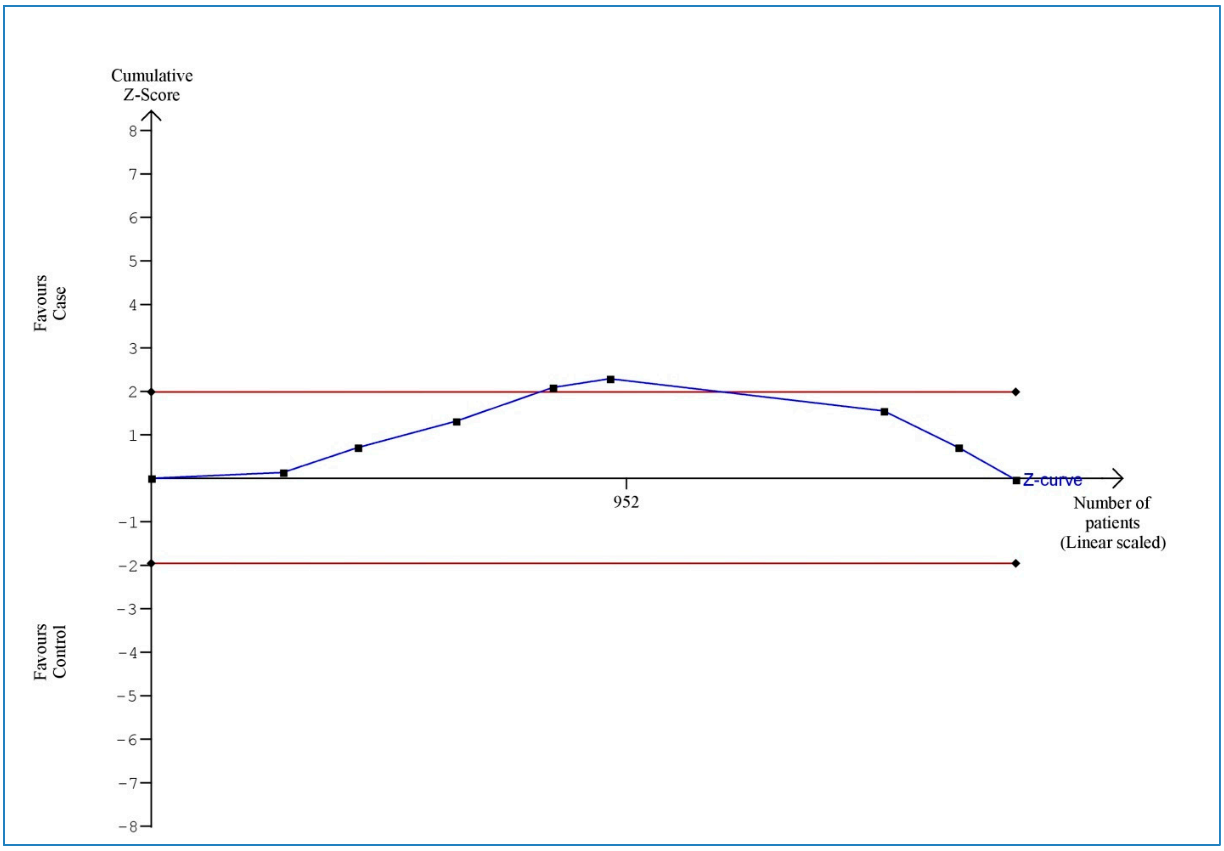

Figure S3: Trial sequential analysis of the association of *FokI* polymorphism and the risk of lumbar disk herniation in heterozygous model (D^2: 67%)

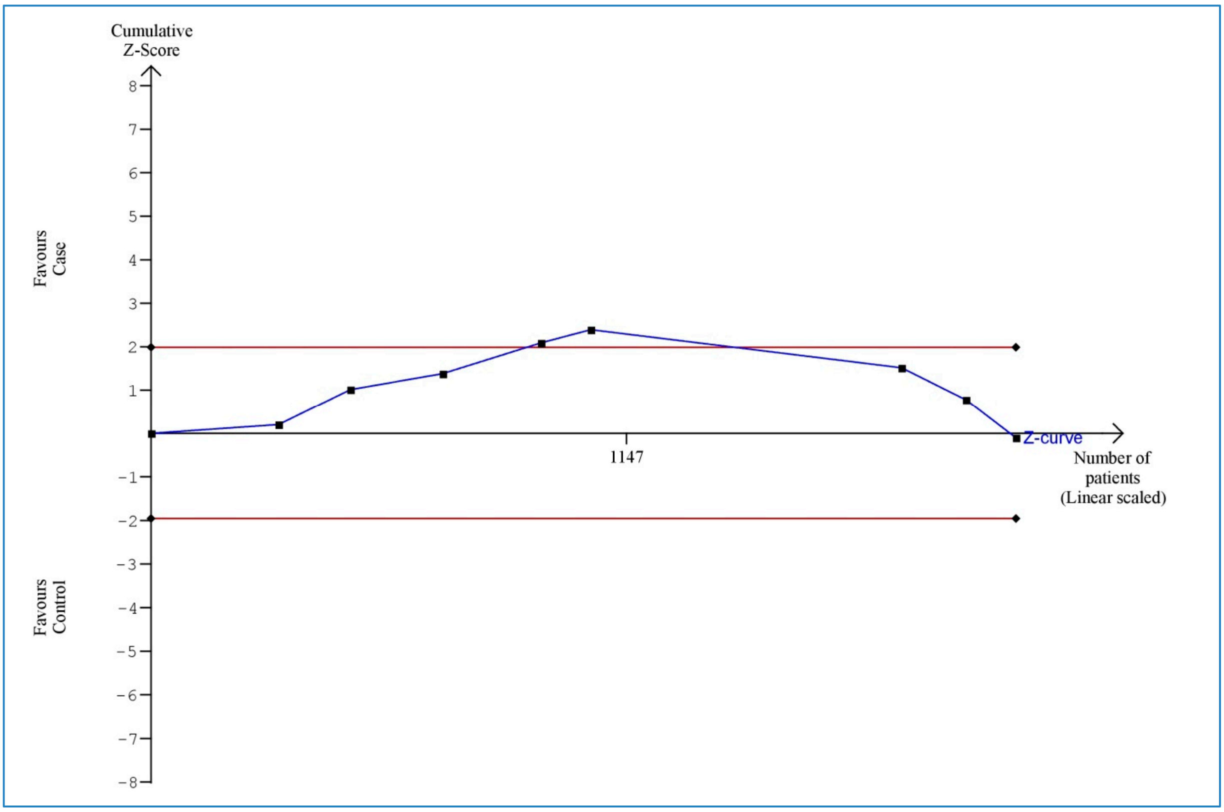

Figure S4: Trial sequential analysis of the association of *FokI* polymorphism and the risk of lumbar disk herniation in dominant model (D^2: 72%)

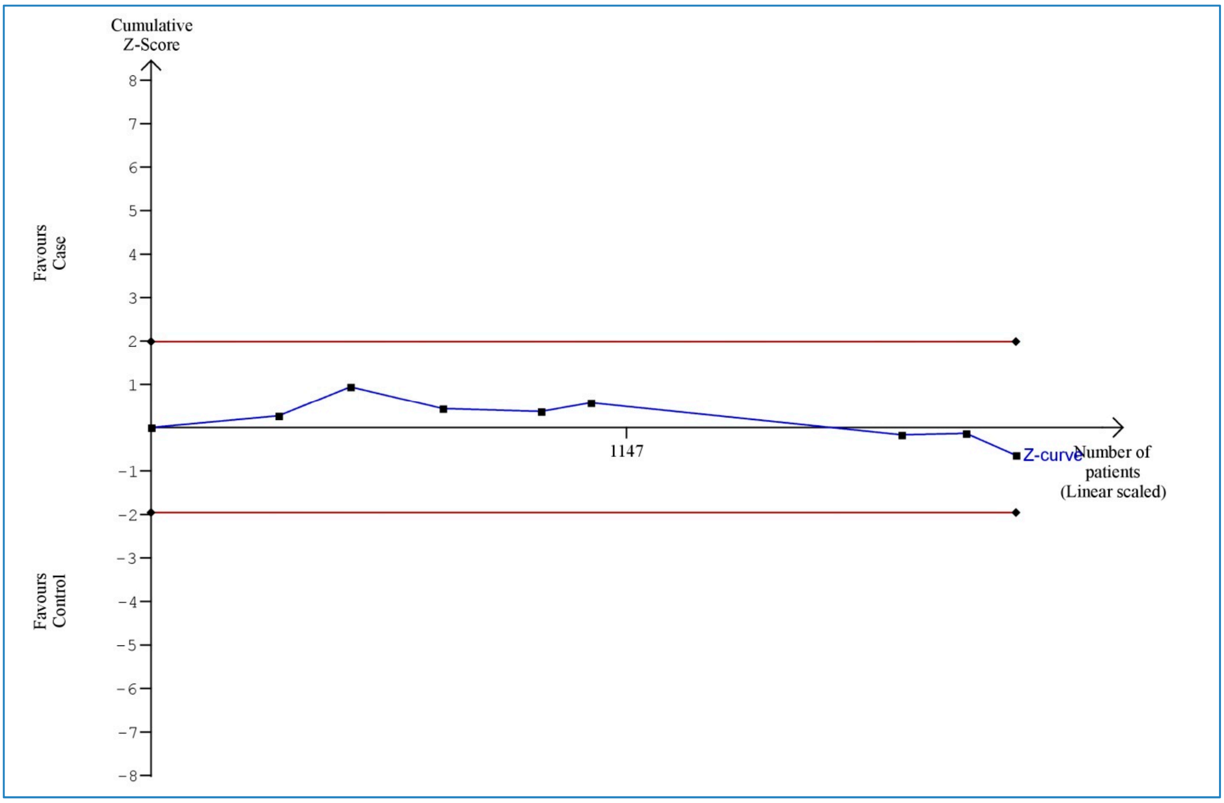

Figure S5: Trial sequential analysis of the association of *FokI* polymorphism and the risk of lumbar disk herniation in recessive model (D^2: 30%)

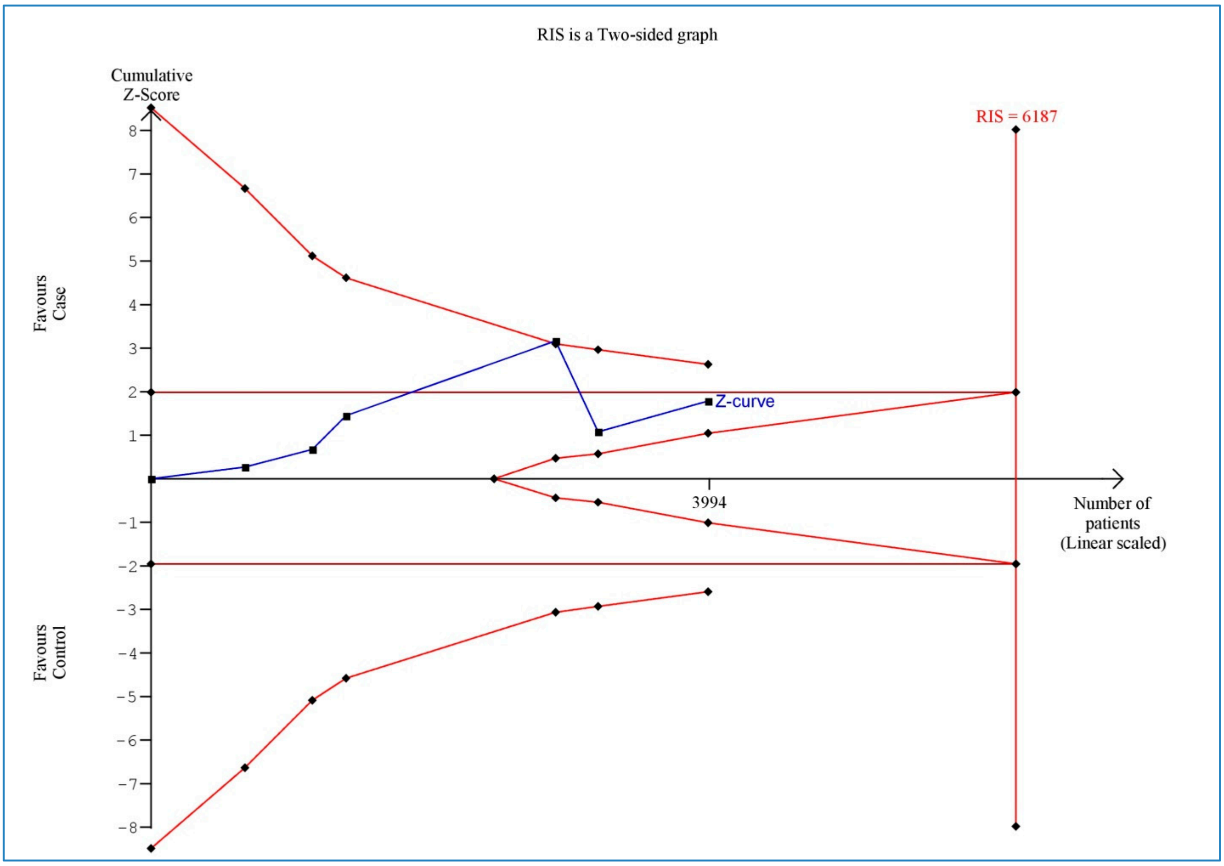

Figure S6: Trial sequential analysis of the association of *TaqI* polymorphism and the risk of lumbar disk herniation in allelic model (D^2: 67%)

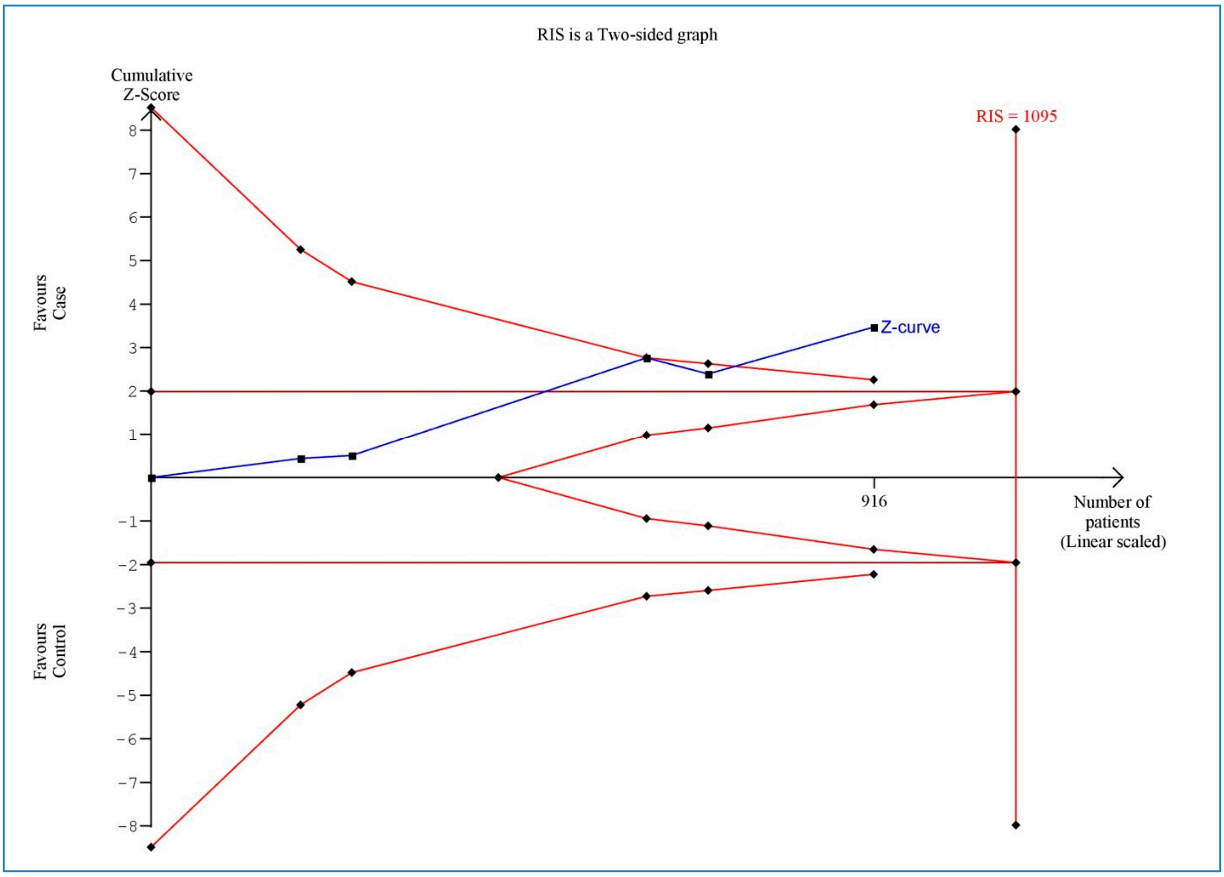

Figure S7: Trial sequential analysis of the association of *TaqI* polymorphism and the risk of lumbar disk herniation in homozygous model (D^2: 44%)

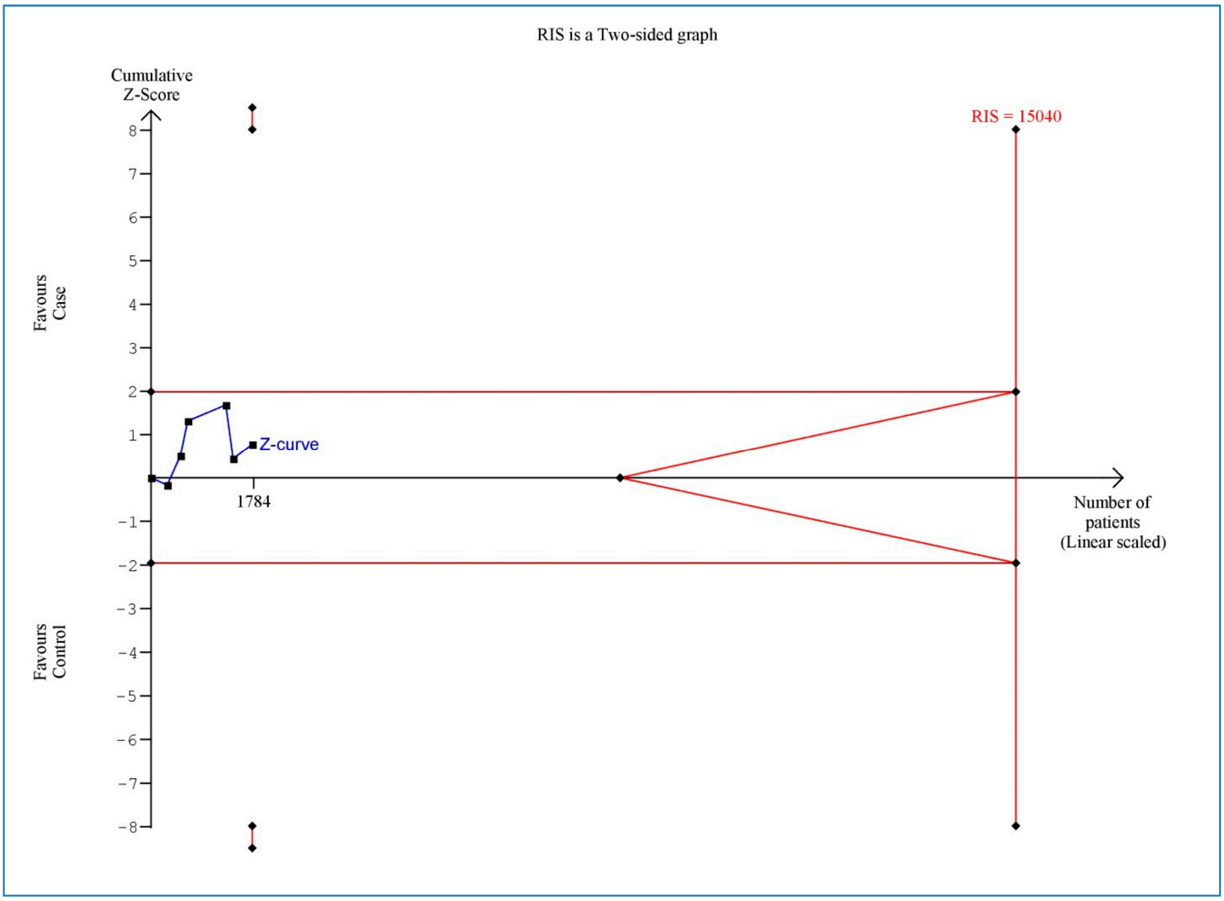

Figure S8: Trial sequential analysis of the association of *TaqI* polymorphism and the risk of lumbar disk herniation in heterozygous model (D^2: 81%)

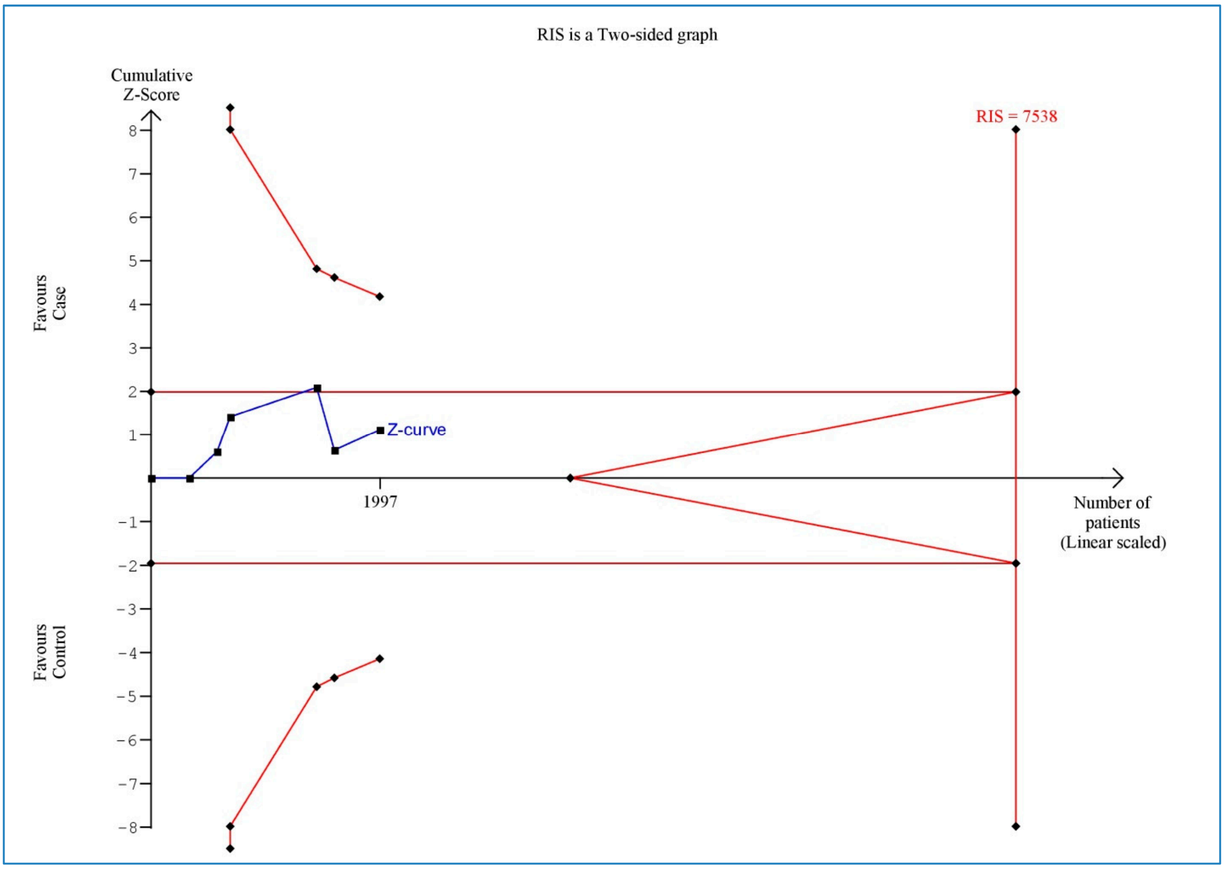

Figure S9: Trial sequential analysis of the association of *TaqI* polymorphism and the risk of lumbar disk herniation in dominant model (D^2: 79%)

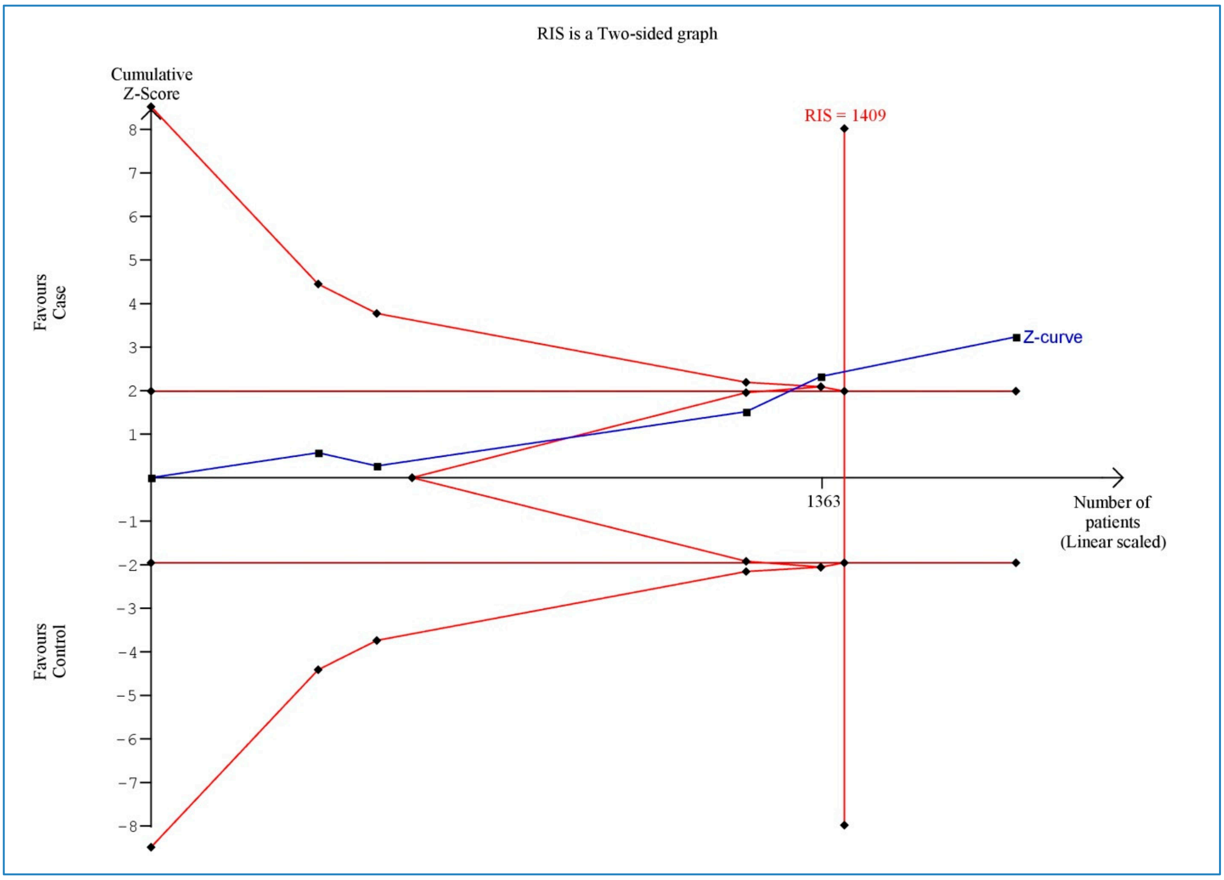

Figure S10: Trial sequential analysis of the association of *TaqI* polymorphism and the risk of lumbar disk herniation in recessive model (D^2: 0%)
