# Supplementary material for: Association of VDR Polymorphisms (FokI, ApaI, and TaqI) with Susceptibility to Lumbar Disc Herniation: Systematic Review, Meta-Analysis, Trial Sequential Analysis, and Transcriptional Prediction
Source: Medicina (Kaunas). 2025 May 12;61(5):882. doi: 10.3390/medicina61050882 (PMC12113608; doi:10.3390/medicina61050882)
Supplement: Supplementary file 1 [file medicina-61-00882-s001.zip › Supplementary File S3.pdf]

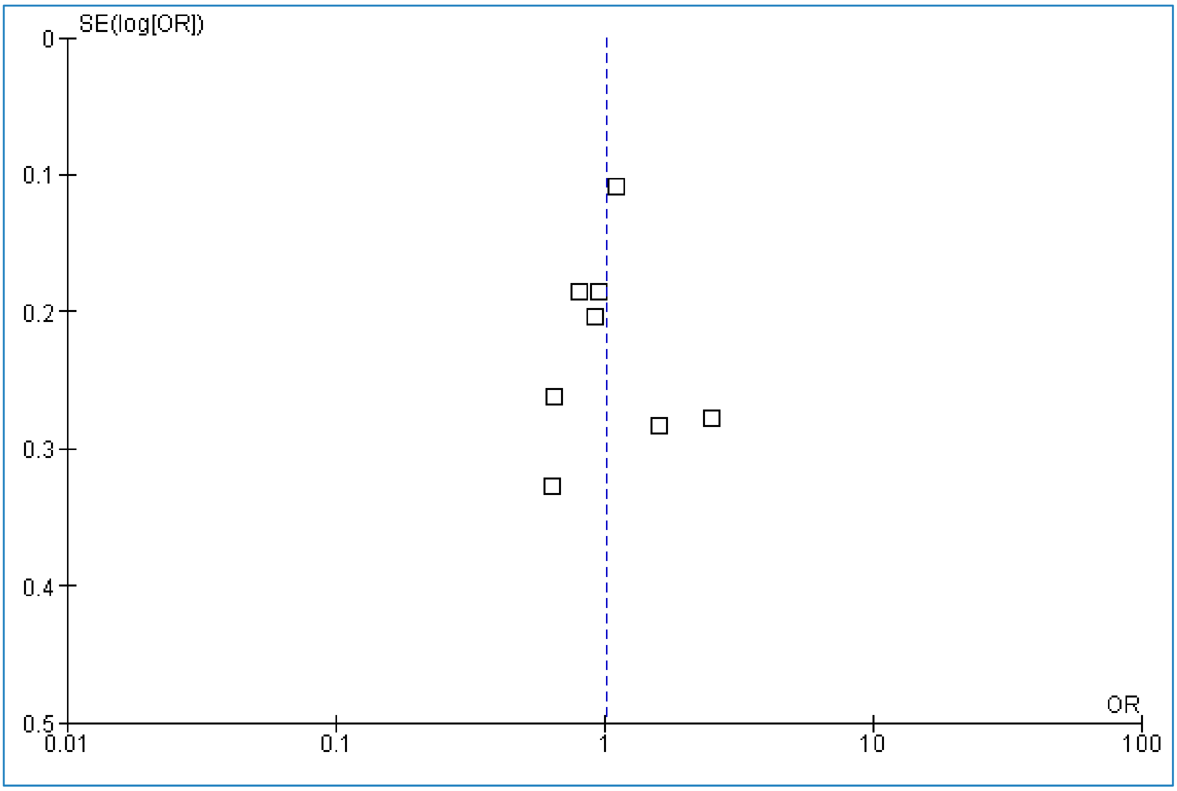

Figure S1: Funnel plot of the association of *FokI* polymorphism and the risk of lumbar disk herniation in allelic model

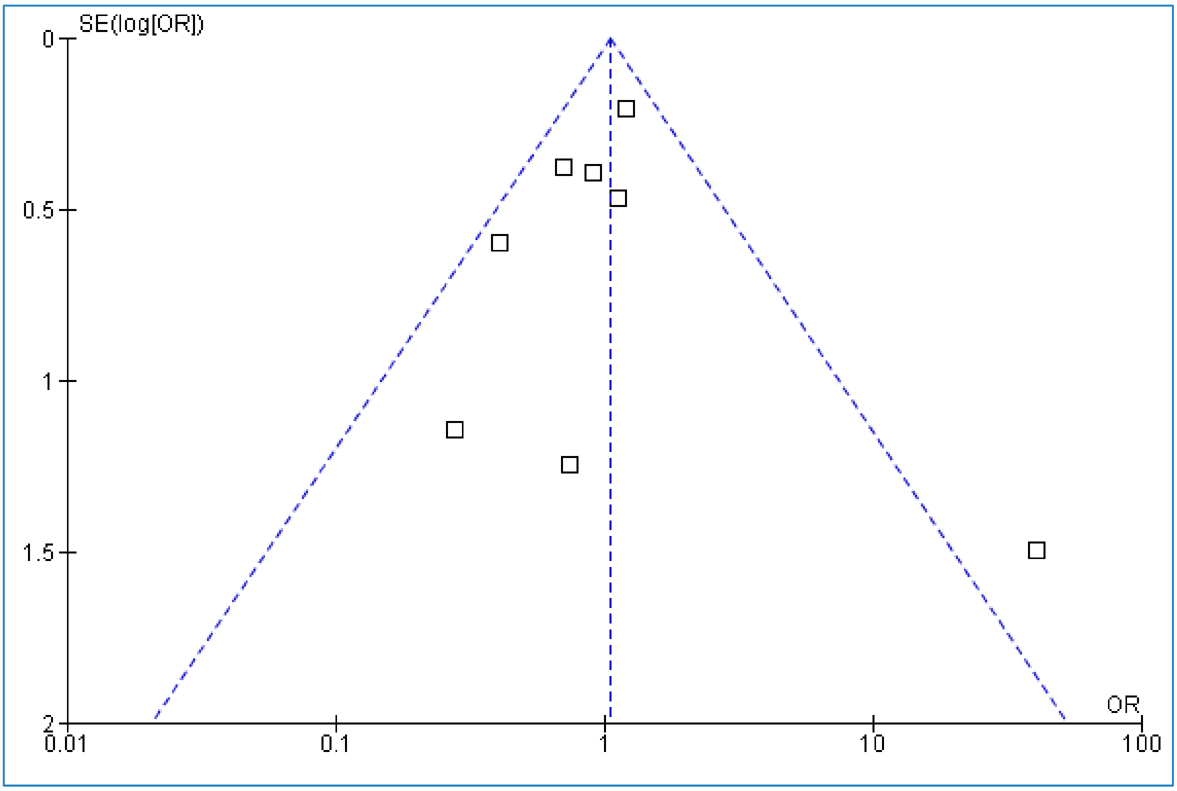

Figure S2: Funnel plot of the association of *FokI* polymorphism and the risk of lumbar disk herniation in homozygous model

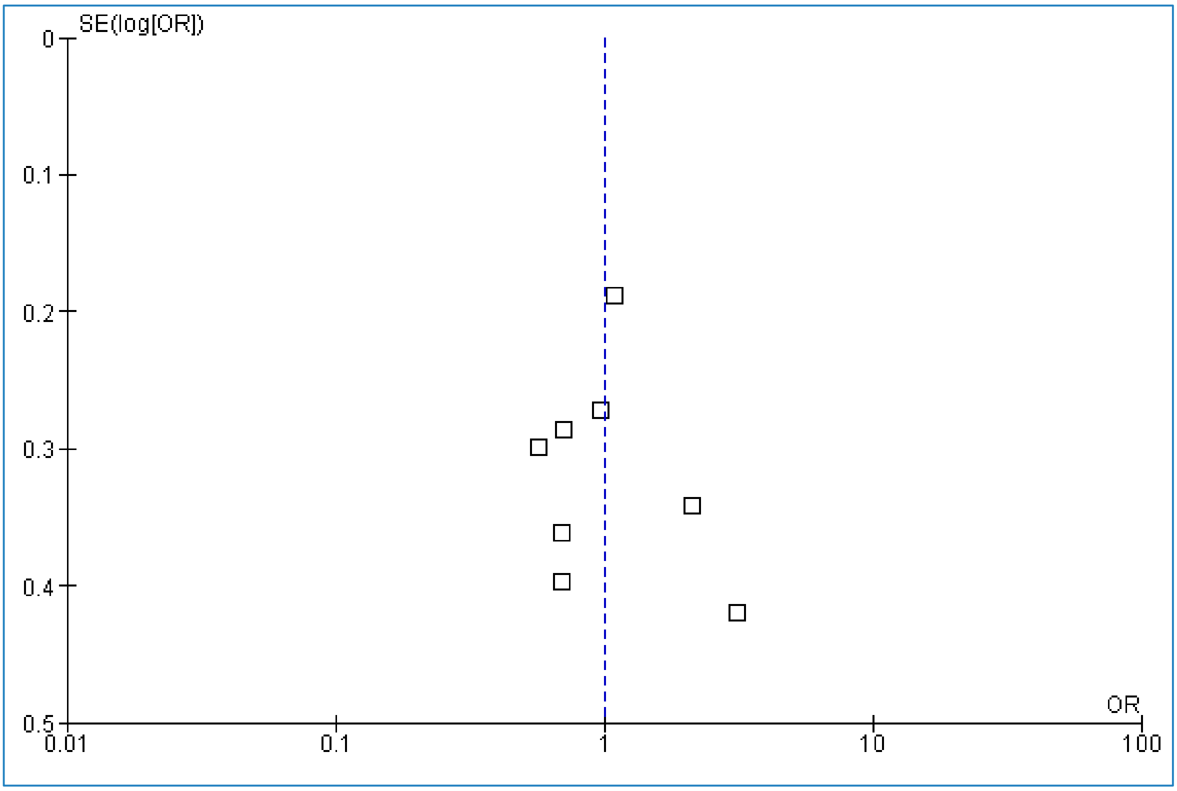

Figure S3: Funnel plot of the association of *FokI* polymorphism and the risk of lumbar disk herniation in heterozygous model

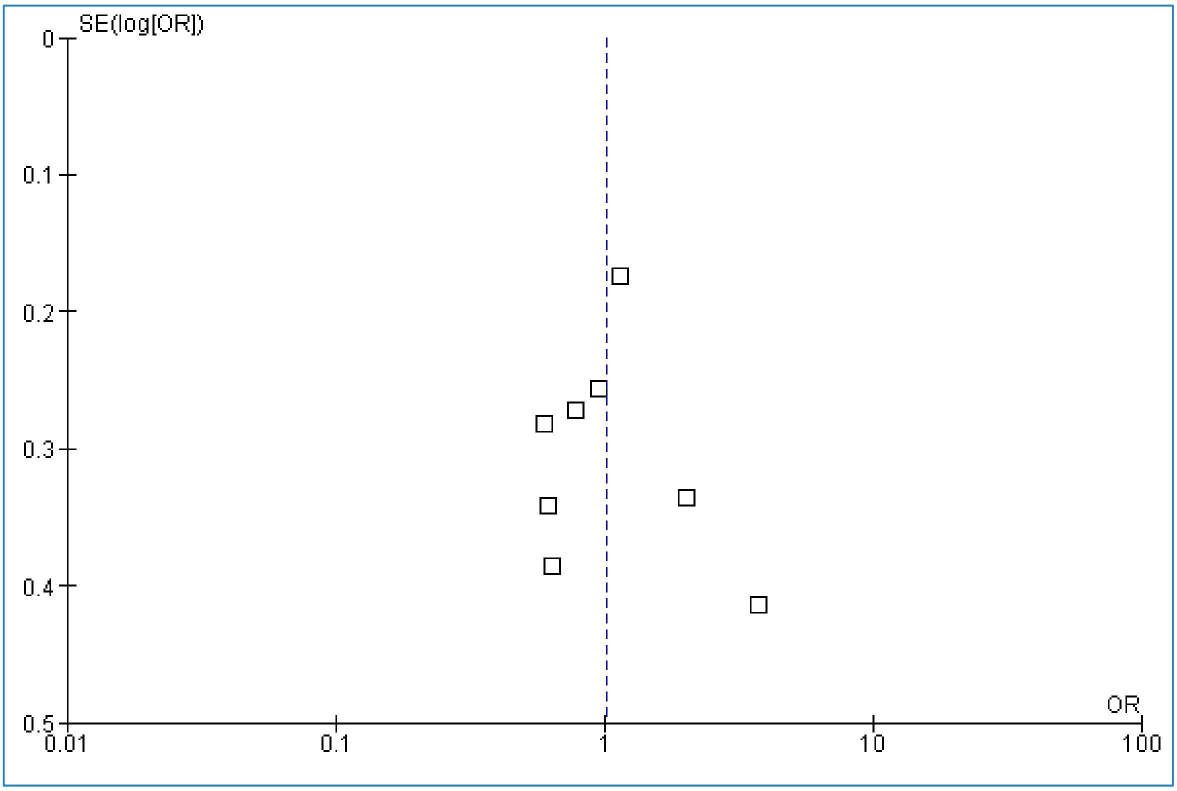

Figure S4: Funnel plot of the association of *FokI* polymorphism and the risk of lumbar disk herniation in dominant model

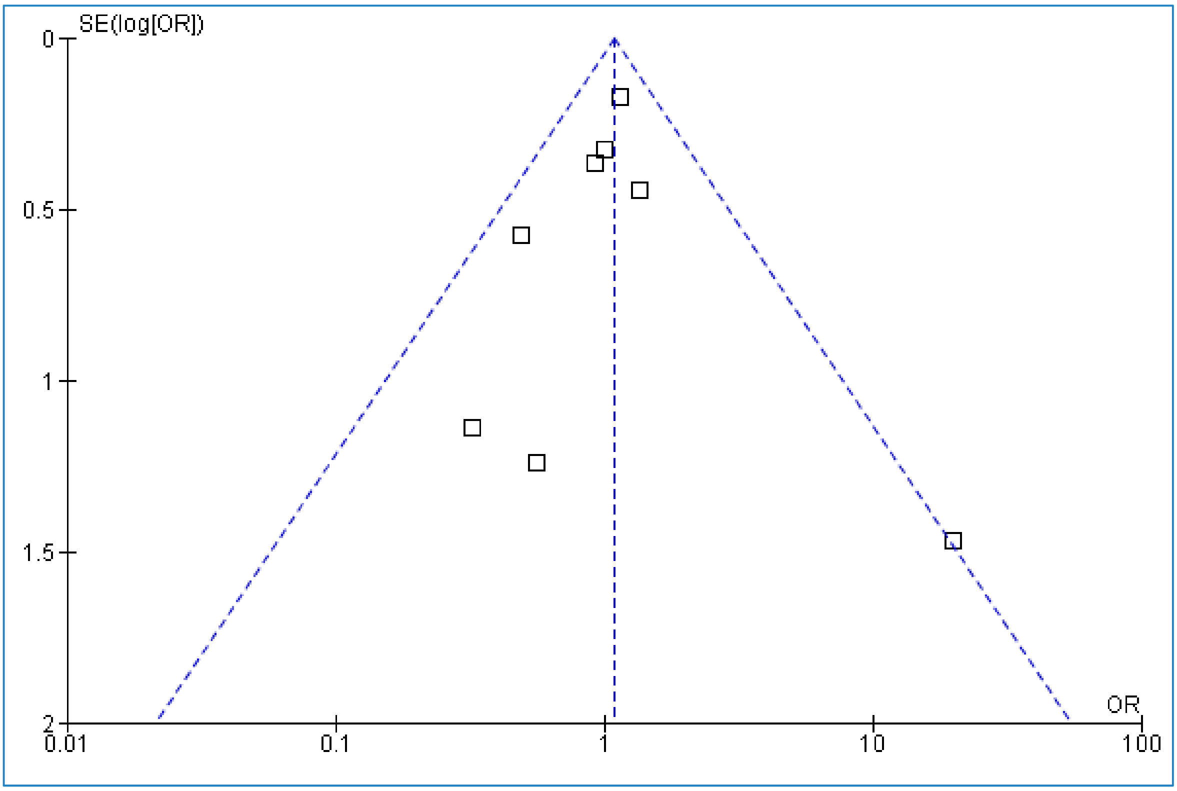

Figure S5: Funnel plot of the association of *FokI* polymorphism and the risk of lumbar disk herniation in recessive model

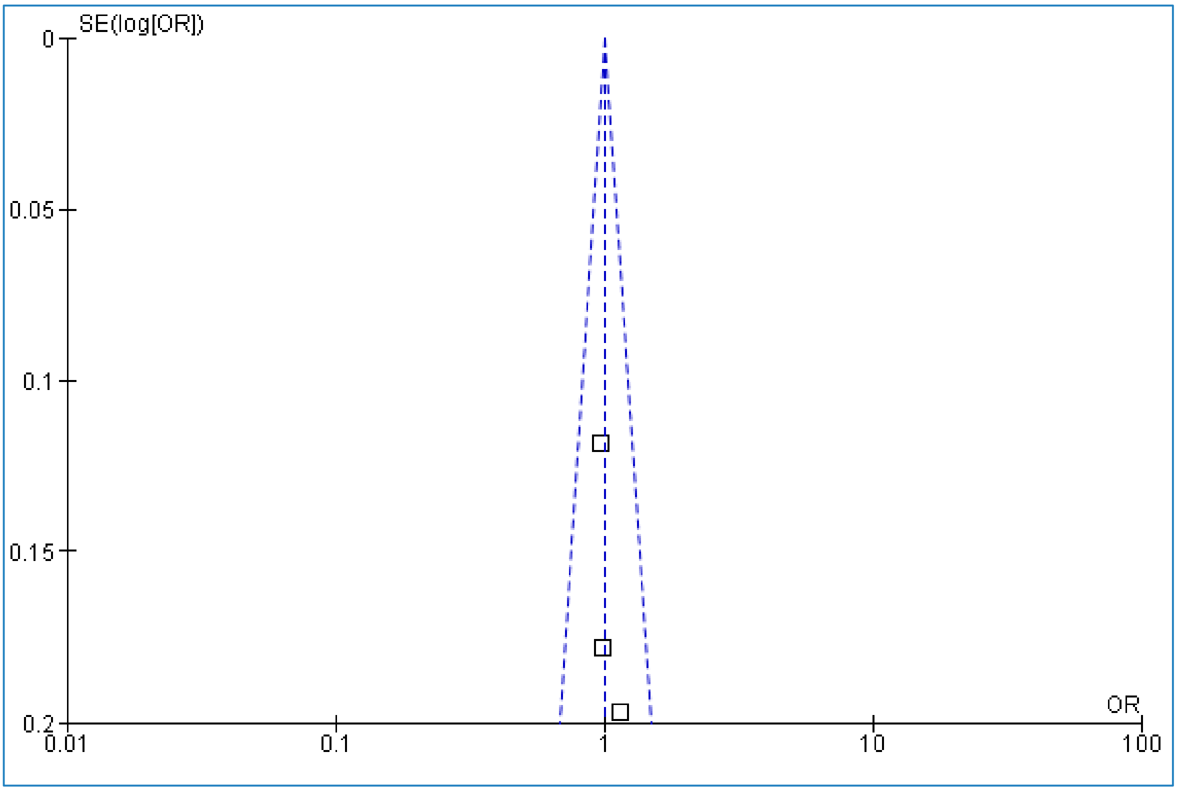

Figure S6: Funnel plot of the association of *ApaI* polymorphism and the risk of lumbar disk herniation in allelic model

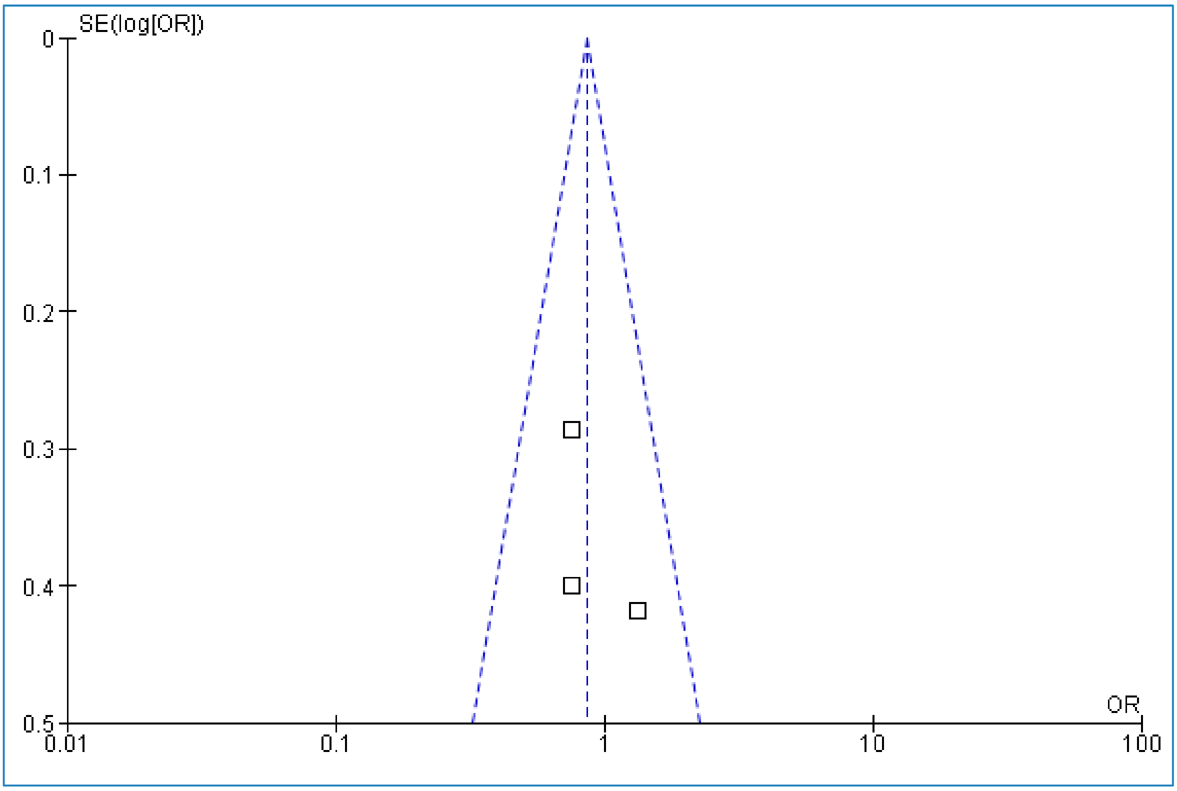

Figure S7: Funnel plot of the association of *ApaI* polymorphism and the risk of lumbar disk herniation in homozygous model

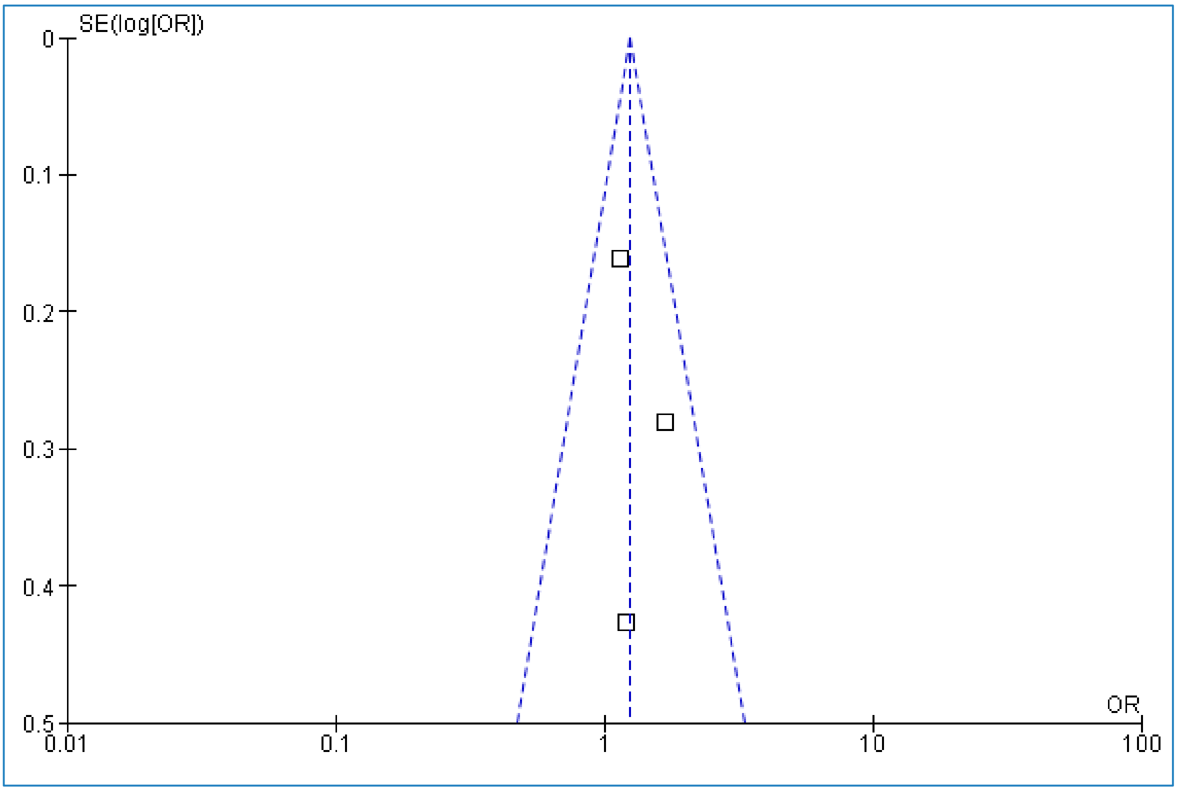

Figure S8: Funnel plot of the association of *ApaI* polymorphism and the risk of lumbar disk herniation in heterozygous model

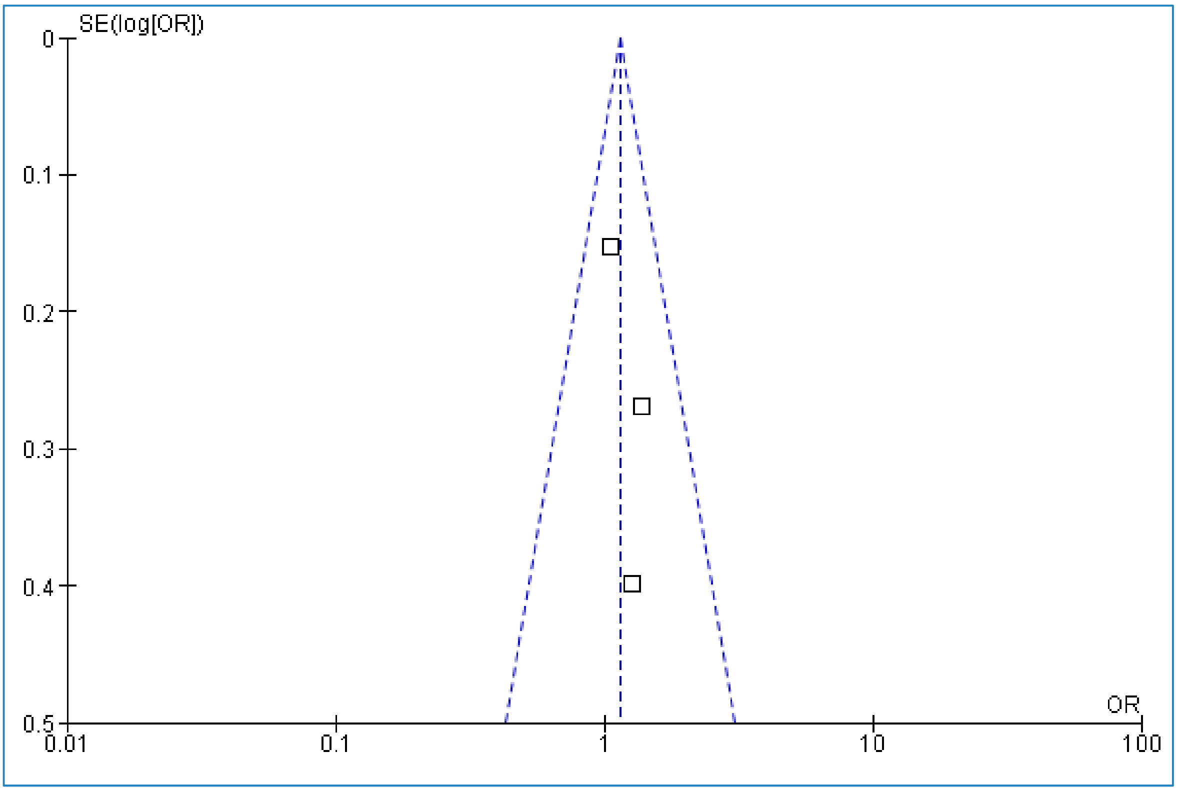

Figure S9: Funnel plot of the association of *ApaI* polymorphism and the risk of lumbar disk herniation in dominant model

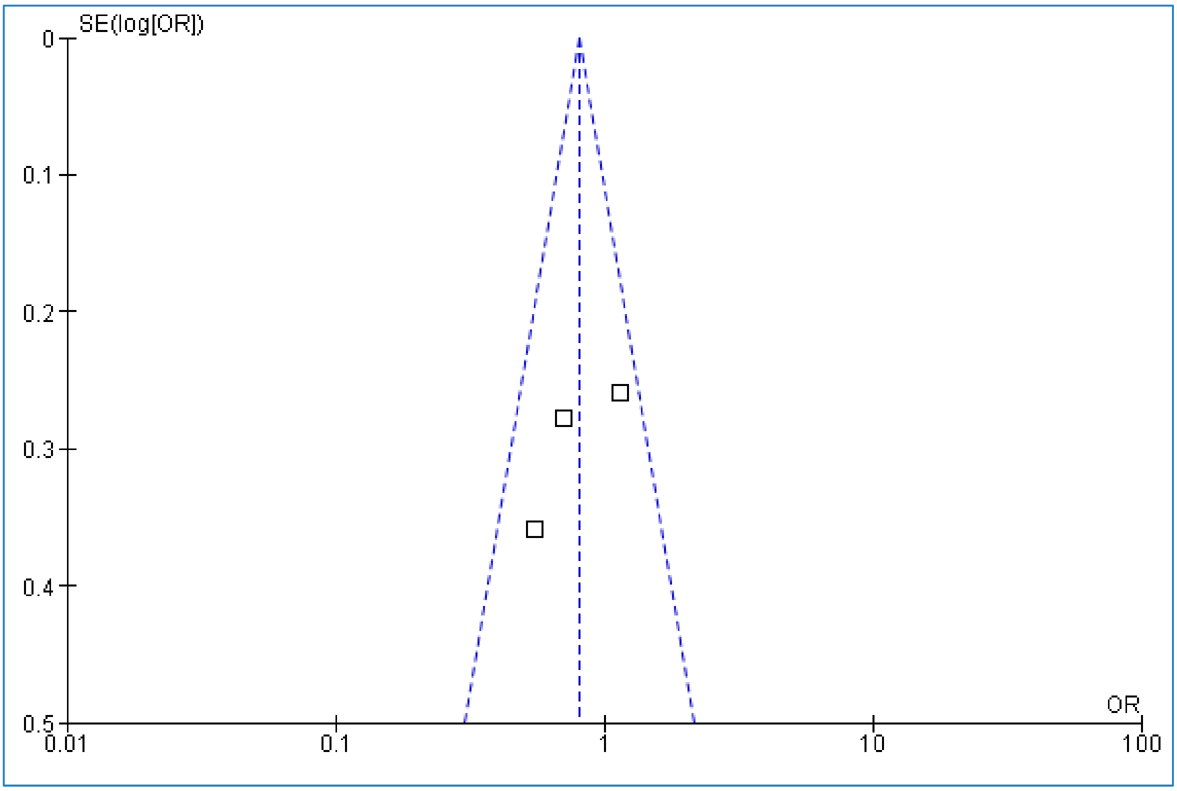

Figure S10: Funnel plot of the association of *ApaI* polymorphism and the risk of lumbar disk herniation in recessive model

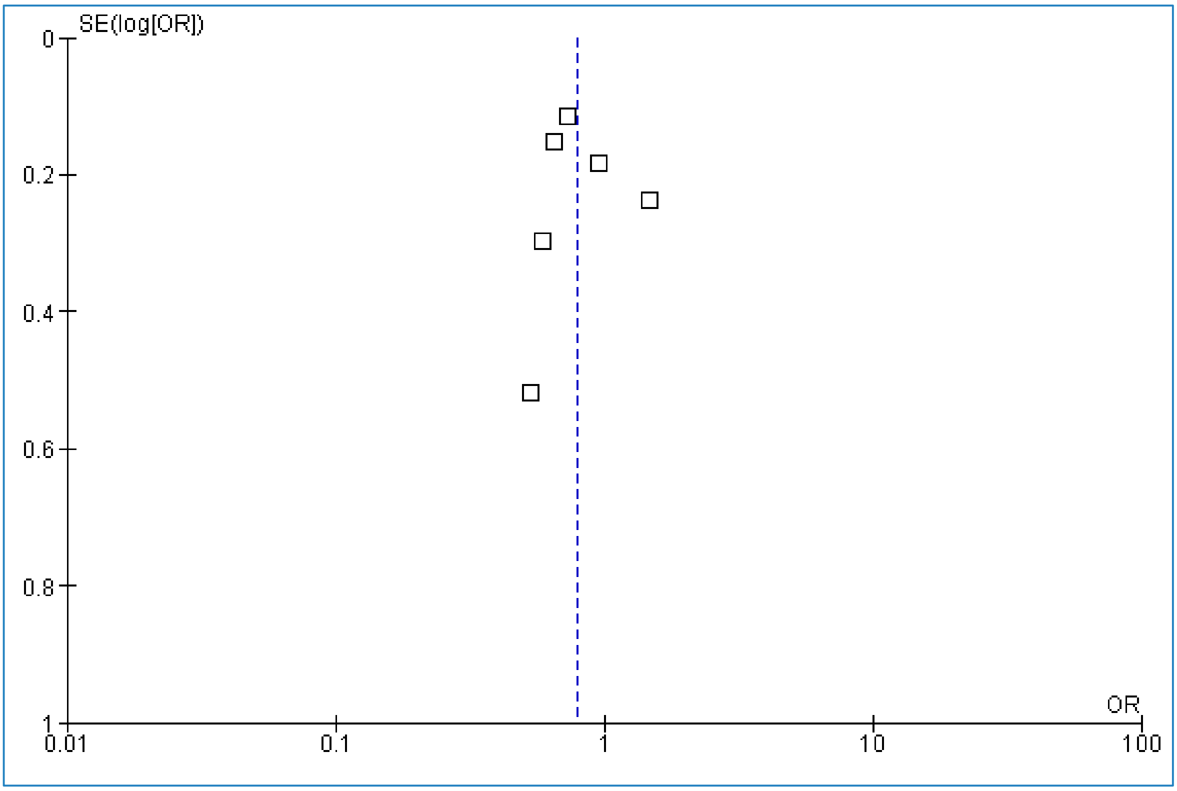

Figure S11: Funnel plot of the association of *TaqI* polymorphism and the risk of lumbar disk herniation in allelic model

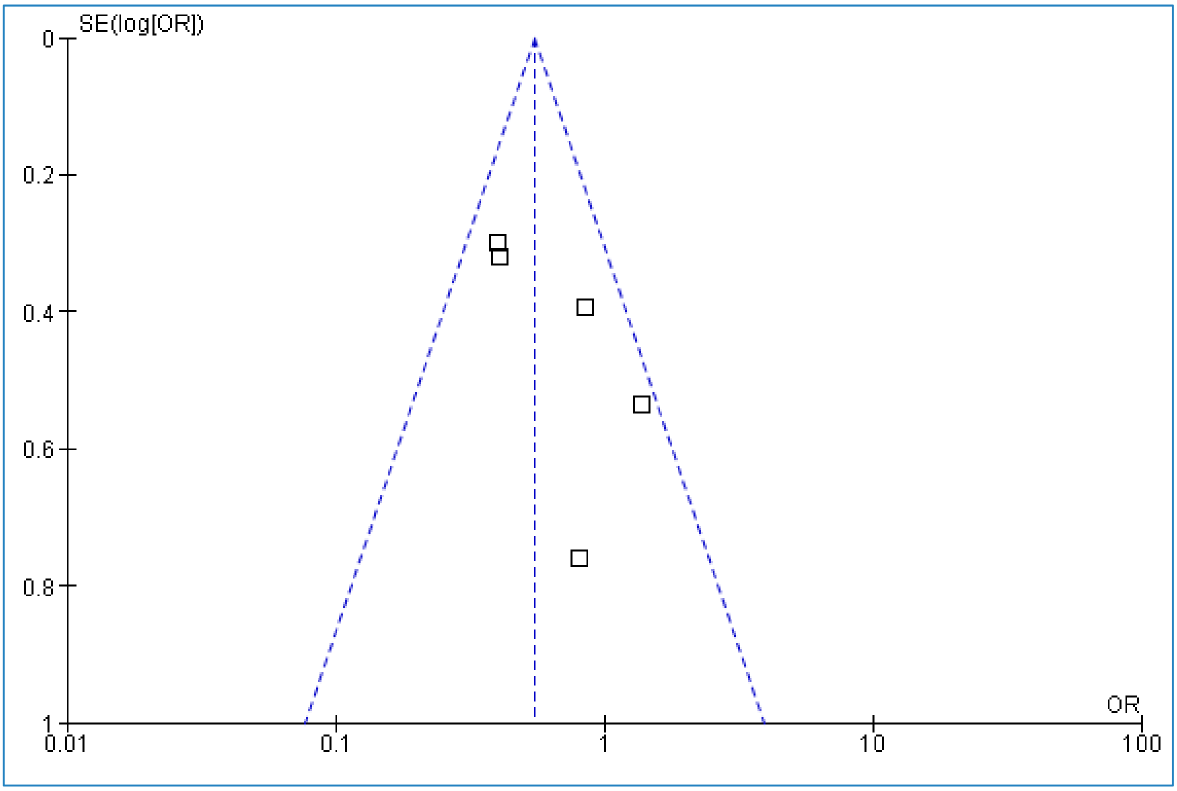

Figure S12: Funnel plot of the association of *TaqI* polymorphism and the risk of lumbar disk herniation in homozygous model

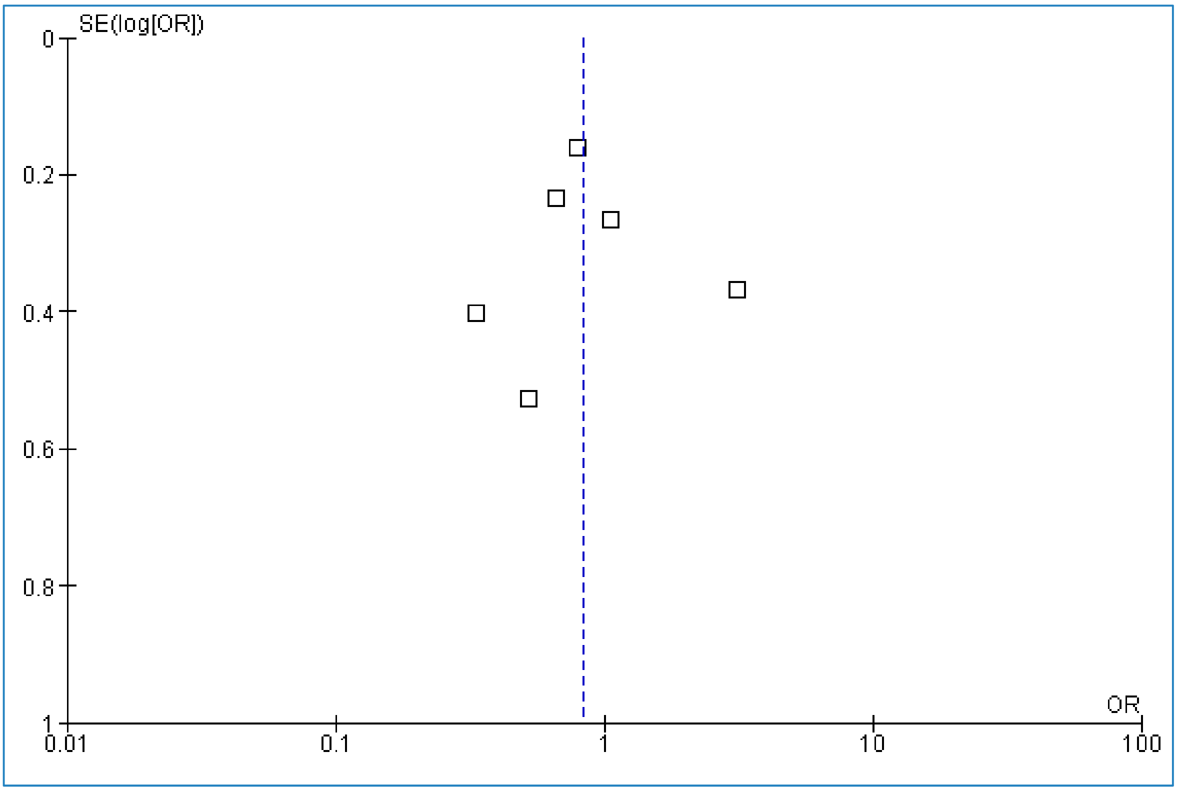

Figure S13: Funnel plot of the association of *TaqI* polymorphism and the risk of lumbar disk herniation in heterozygous model

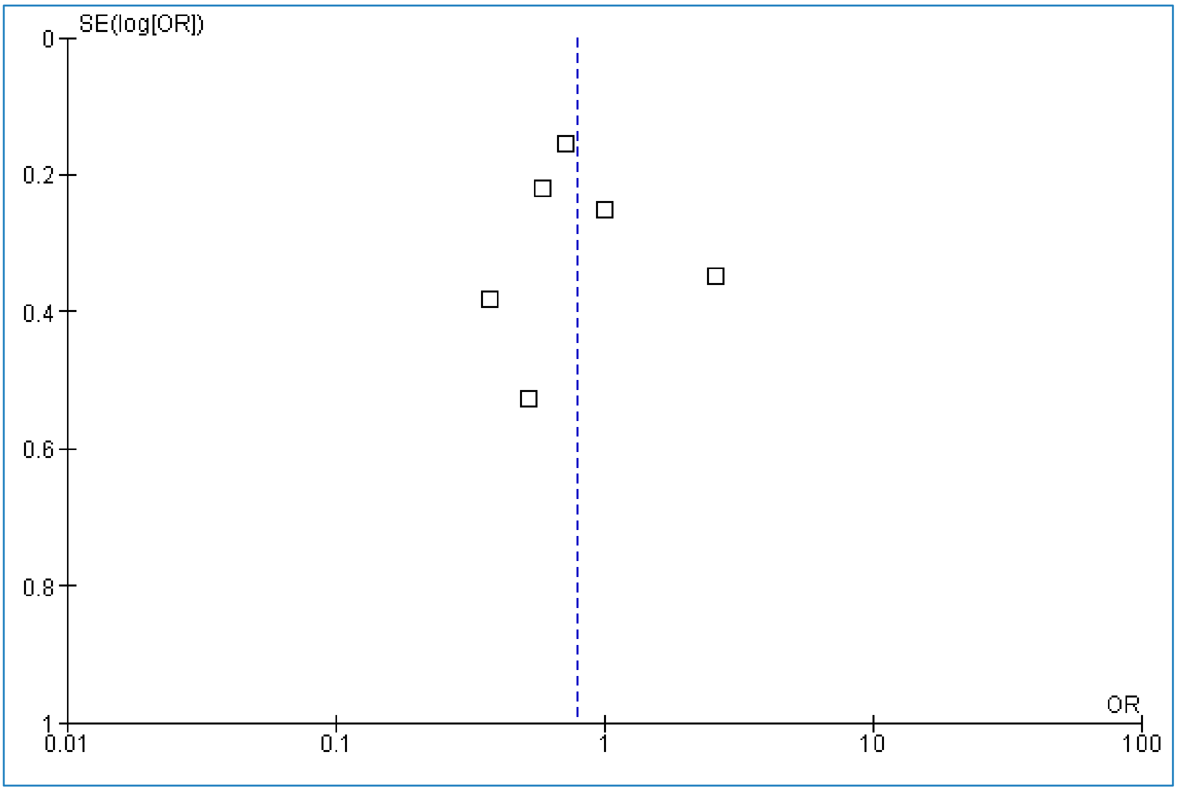

Figure S14: Funnel plot of the association of *TaqI* polymorphism and the risk of lumbar disk herniation in dominant model

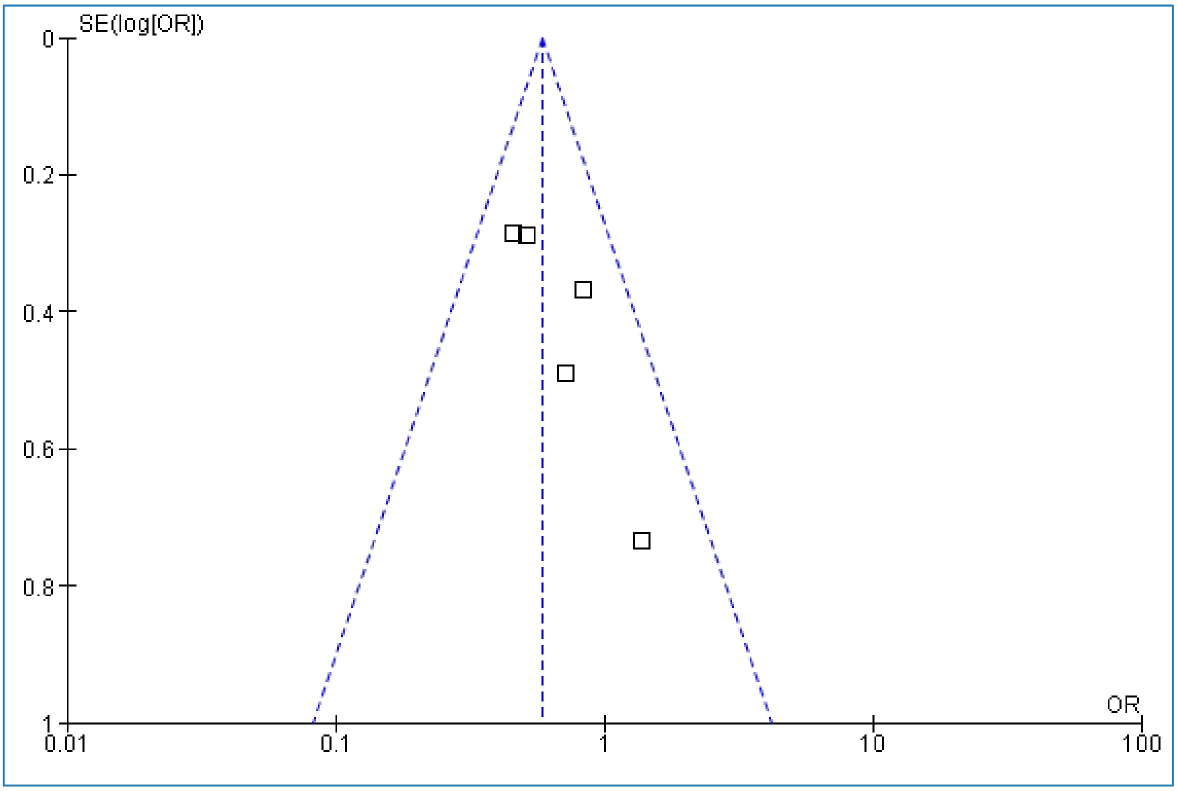

Figure S15: Funnel plot of the association of *TaqI* polymorphism and the risk of lumbar disk herniation in recessive model
